# Segmented Flow Processes to Overcome Hurdles of Whole‐Cell Biocatalysis in the Presence of Organic Solvents

**DOI:** 10.1002/anie.202015887

**Published:** 2021-06-10

**Authors:** Niklas Adebar, Alina Nastke, Jana Löwe, Harald Gröger

**Affiliations:** ^1^ Chair of Industrial Organic Chemistry and Biotechnology Faculty of Chemistry Bielefeld University Universitätsstr. 25 33615 Bielefeld Germany

**Keywords:** biocatalysis, biphasic reaction, flow chemistry, segmented flow processes, whole-cell catalysis

## Abstract

In modern process development, it is imperative to consider biocatalysis, and whole‐cell catalysts often represent a favored form of such catalysts. However, the application of whole‐cell catalysis in typical organic batch two‐phase synthesis often struggles due to mass transfer limitations, emulsion formation, tedious work‐up and, thus, low yields. Herein, we demonstrate that utilizing segmented flow tools enables the conduction of whole‐cell biocatalysis efficiently in biphasic media. Exemplified for three different biotransformations, the power of such segmented flow processes is shown. For example, a 3‐fold increase of conversion from 34 % to >99 % and a dramatic simplified work‐up leading to a 1.5‐fold higher yield from 44 % to 65 % compared to the analogous batch process was achieved in such a flow process.

## Graphical abstract

Utilizing tools of segmented flow chemistry enables the conduction of whole‐cell biocatalysis efficiently in biphasic media. Exemplified for different biotransformations with an imine reductase, aldoxime dehydratase, allene oxide synthase and cyclase, the power of flow processes is demonstrated, leading to a 3‐fold increase of conversion as well as a dramatic simplified work‐up and a 1.5‐fold higher yield compared to the analogous batch processes.

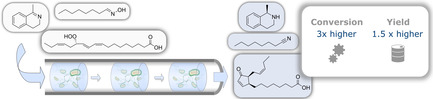

## Introduction

**Figure 1:**
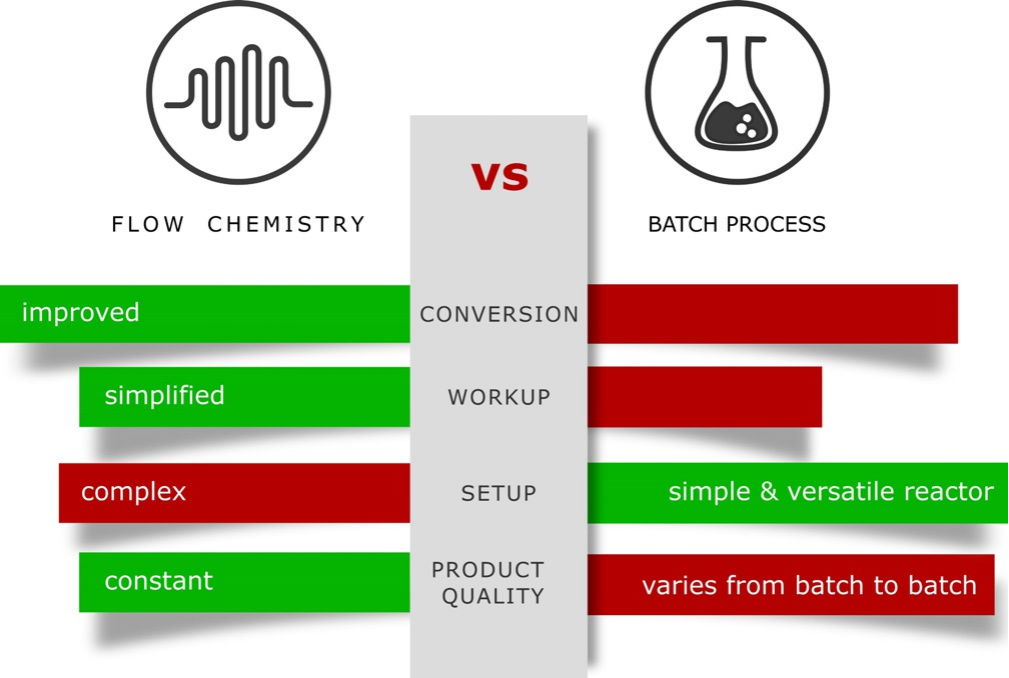
General comparison of (continuous) flow versus batch chemistry.

The application of biocatalysis in organic synthesis has improved substantially over the last decades leading to many successes.[^1^, ^2^] However, some issues remained unsolved since a long time. In particular, the incompatibility of many biocatalytic systems with various organic solvents still represents a major limitation in synthesis and process design.[^3^, ^4^] The challenge to benefit from organic solvents through an improved substrate availability while at the same time avoiding its deactivation of the biocatalyst has been addressed by us by means of flow chemistry techniques. Herein we report on a system for improved whole‐cell biocatalysis based on liquid‐liquid segmented flow, which remarkably overcomes the known limitations when using analogous liquid‐liquid systems in the batch mode (Figure 1). With respect to such flow processes it should be added that several years ago the U.S. Food and Drug Agency (FDA) as well as the European Medicines Agency (EMA) added a recommendation for continuous manufacturing to their guidelines.^[5]^

In particular, constant product quality without human intervention was pointed out as important for future process development. In addition, these features are important not only in pharmaceutical chemistry,[^6^, ^7^, ^8^] as the bulk chemistry field can also benefit from flow processing. Increased heat and mass transfer as well as high safety for toxic or explosive compounds are general benefits.[^9^, ^10^]

**Figure 2:**
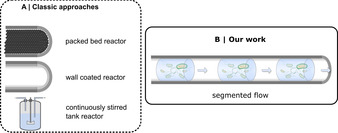
Scheme of the developed organic/aqueous segmented flow system utilizing whole‐cell catalysts compared to classic approaches for continuous biocatalysis utilising whole‐cells.

However, in spite of its tremendous application potential, so far most continuous processes involving biocatalysis have been limited to the application of lipases or alcohol dehydrogenases (ADH).[^11^, ^12^, ^13^] Due to their extreme resistance against temperature and organic solvents as well as their commercial availability in immobilised form, many processes involving lipases have been developed.[^14^, ^15^] Recently, flow processes have also been developed for many other biocatalytic systems.[^16^, ^17^, ^18^] In contrast, only few examples of continuous flow processes with whole‐cell catalysts have been reported.^[19]^ To the best of our knowledge, all reported systems rely exclusively on the combination of immobilised cells in packed bed reactors,[^20^, ^21^, ^22^, ^23^, ^24^] catalytic biofilms,[^25^, ^26^] wall coated reactors^[27]^ or hydrogel‐immobilized cells in segmented flow.^[28]^ These concepts are illustrated in Figure 2.

Buehler et al. showed that mass‐transfer limited reactions using isolated and purified enzymes can benefit from segmented flow systems.[^29^, ^30^] This work centers on an ADH from *Lactobacillus brevis* for reducing heptanal to the corresponding alcohol.^[29]^ Thereafter, only a few further biphasic biocatalytic flow processes were reported.^[31]^ Most recently, the Wirth group successfully applied biocatalysis in a high performance counter current chromatography (HPCCC) device, which enhanced the mass transfer immensely.^[32]^

**Scheme 1:**
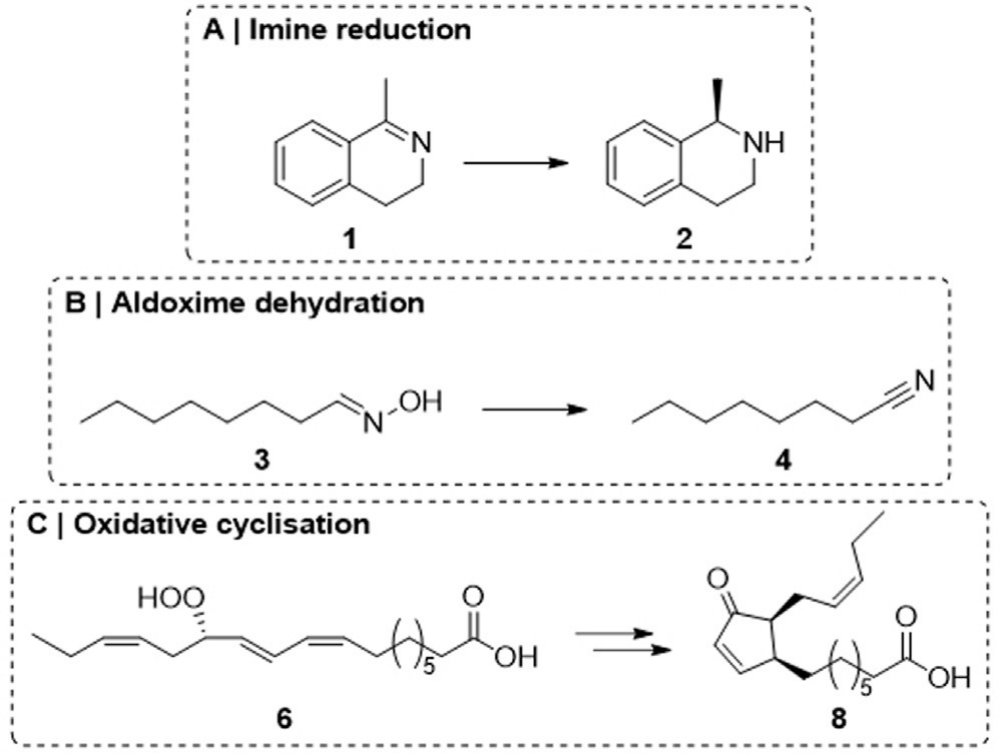
Investigated model processes with whole‐cells: A) IRED‐catalysed reduction of 1‐methyl‐3,4‐dihydroisoquinoline (**1**) to the corresponding amine (*R*)‐**2**, B) OxdB‐catalysed dehydration of *n*‐octanal oxime (**3**) to *n*‐octanenitrile (**4**) and C) Oxidative cyclisation towards 12‐OPDA (**8**) starting from 13‐(*S*)‐hydroperoxylinolenic acid (**6**).

In our work presented here, we investigated the impact of such segmented flow systems on a variety of whole‐cell based systems, which revealed different advantages. Besides the aldoxime dehydratase (Oxd)‐catalysed synthesis of *n*‐octanenitrile (**4**), which is used as bulk chemical,[^33^, ^34^, ^35^] the preparation of 12‐oxophytodienoic acid (12‐OPDA, **8**), a complex chiral plant hormone intermediate,^[36]^ using a whole‐cell catalyst containing an allene oxide synthase and cyclase was investigated. Along with these examples, a cofactor dependent imine reductase (IRED) was used for the synthesis of chiral cyclic amines (see Scheme 1). For all experiments with these whole‐cell catalysts, a segmented flow system was applied and compared to a biphasic batch approach. It is noteworthy that in each of these three whole‐cell processes a different current challenge in the field of biocatalysis, e.g., phase separation of emulsions as well as mass transfer issues, has been successfully addressed. In detail, different stirring rates in the batch mode and different flow rates in the flow mode were investigated and compared for the IRED‐catalysed reduction. The impact of solvent additives in the batch versus flow mode was investigated for the oxime dehydration using OxdB as a highly solvent labile biocatalyst. In addition, the efficiency of product isolation was studied in flow and compared to the batch mode exemplified for the particularly challenging example of the 12‐OPDA synthesis, since 12‐OPDA is an emulsifier.

## Results and Discussion

### Imine Reduction in a Continuous Segmented Flow Mode: The Impact of Organic Solvents and Studies on Mass Transfer

As a first example we chose the enantioselective reduction of the C=N‐double bond of 1‐methyl‐3,4‐dihydroisoquinoline (**1**) to the corresponding amine (*R*)‐**2** as a model reaction for the biocatalytic synthesis of cyclic amines. The reaction was carried out using an IRED with NADPH as cofactor. The resulting oxidized species of the cofactor was regenerated in situ using d‐glucose and glucose dehydrogenase (GDH) under formation of one equivalent of gluconolactone, which irreversibly opens up to gluconic acid and therefore moves the equilibrium of the reaction towards the product side. For this process, a recombinant *E. coli* whole‐cell catalyst containing an IRED from *Streptomyces viridochromogenes* and a GDH from *Bacillus subtillis* in overexpressed form, recently developed in our research group,^[37]^ was used. Initially, we investigated the reaction system in a batch mode in order to obtain a benchmark and to gain insight into some important reaction parameters, such as choice of organic solvent and stirring rate. The solvent can have a tremendous influence on the reaction system, as it affects compound solubility and distribution as well as cell and/or enzyme deactivation. As substrate solubility and cell deactivation have no correlation with each other, predicting the solvent of choice represents a challenging task. Thus, for our evaluation on the impact of solvent and stirring rate on the whole‐cell catalyzed imine reduction we chose several water‐immiscible solvents, ranging from highly non‐polar solvents such as cyclohexane to less non‐polar solvents such as methyl *tert*‐butyl ether (MTBE) and 2‐methyltetrahydrofuran (MeTHF). We used preferably less hazardous solvent options, e.g., cyclohexane rather than hexane or pentane. We also avoided the use of chlorinated solvents due to their negative environmental impact and known incompatibility with whole‐cell catalysts. The experiments were conducted at a typical whole‐cell catalyst loading (2 mg_dcm_ mL^−1^ of dry cell mass per overall volume) and a stirring rate of 850 rpm. At first, the substrate (40 mM) was dissolved in the organic solvent, and then added to a KP_i_ buffer solution containing d‐glucose, NADP^+^, methanol (2 vol%) and *E. coli* BL21(DE3) whole‐cells with IRED and GDH therein.

**Figure 3:**
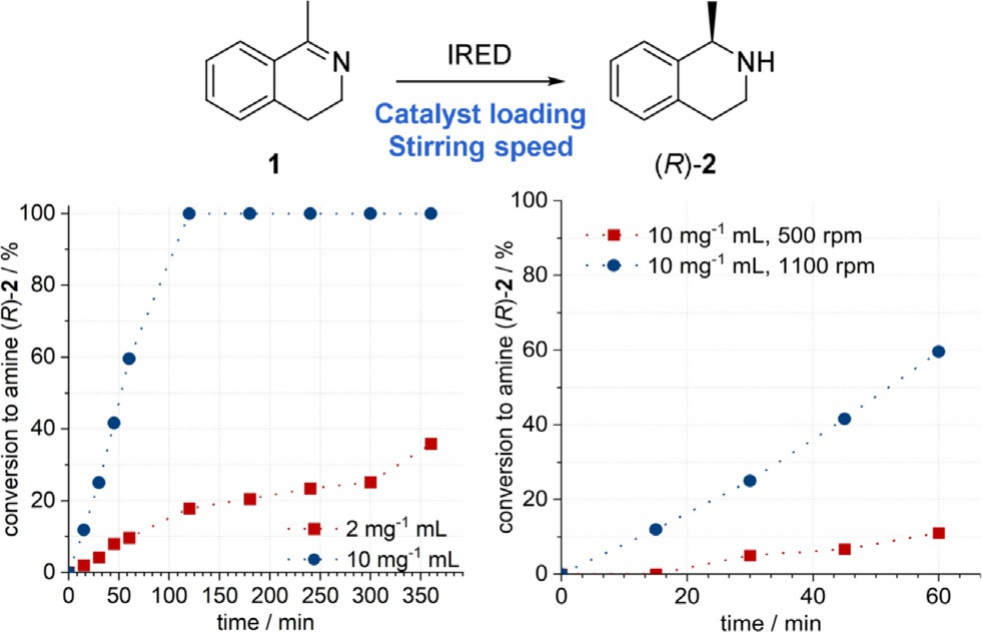
Time course measurements of the conversion to amine (*R*)‐**2** at whole‐cell catalyst loadings of 2 mg_dcm_ mL^−1^ and 10 mg_dcm_ mL^−1^ (left) and investigation of different stirring rates (right). Reaction conditions: Biphasic system with 40 mM of substrate **1** in methylcyclohexane and whole‐cell catalyst in different concentrations, 0.2 mM NADP^+^, 240 mM glucose, 2 % MeOH in KP_i_ buffer (50 mM, pH 7); stirred at 30 °C and 500 rpm or 1100 rpm.

The less polar solvents proved to be the best performing ones with sufficient conversions when using cyclohexane, isooctane and methylcyclohexane (see Supporting Information). In contrast, MTBE, MeTHF and ethyl acetate led to the complete deactivation of the biocatalyst and, thus, no conversion was observed. For further experiments we decided to use methylcyclohexane as an organic solvent, as it provided the highest conversion in this initial work. When conducting batch experiments with a magnetic stirrer at a high stirring rate of 1100 rpm for an intensive mixing of the two phases, a conversion of 36 % after 6 h was observed (Figure 3). After optimizing the whole‐cell catalyst loading to 10 mg_dcm_ mL^−1^, the reaction rate to amine (*R*)‐**2** strongly increased reaching a quantitative conversion within only 2 h reaction time.

As mass transfer is often a limiting factor in catalytic reactions, we became interested in how sensitive this biocatalytic reaction is with respect to the stirring rate. Thus, we investigated the reaction using 10 mg_dcm_ mL^−1^ of whole‐cells in batch mode with a lower stirring rate of 500 rpm (at which the phases remained separated). These experiments revealed a strong mass transfer limitation of the reaction as a 6‐fold higher conversion was achieved within 1 h reaction time when increasing the stirring rate (Figure 3).

**Figure 4:**
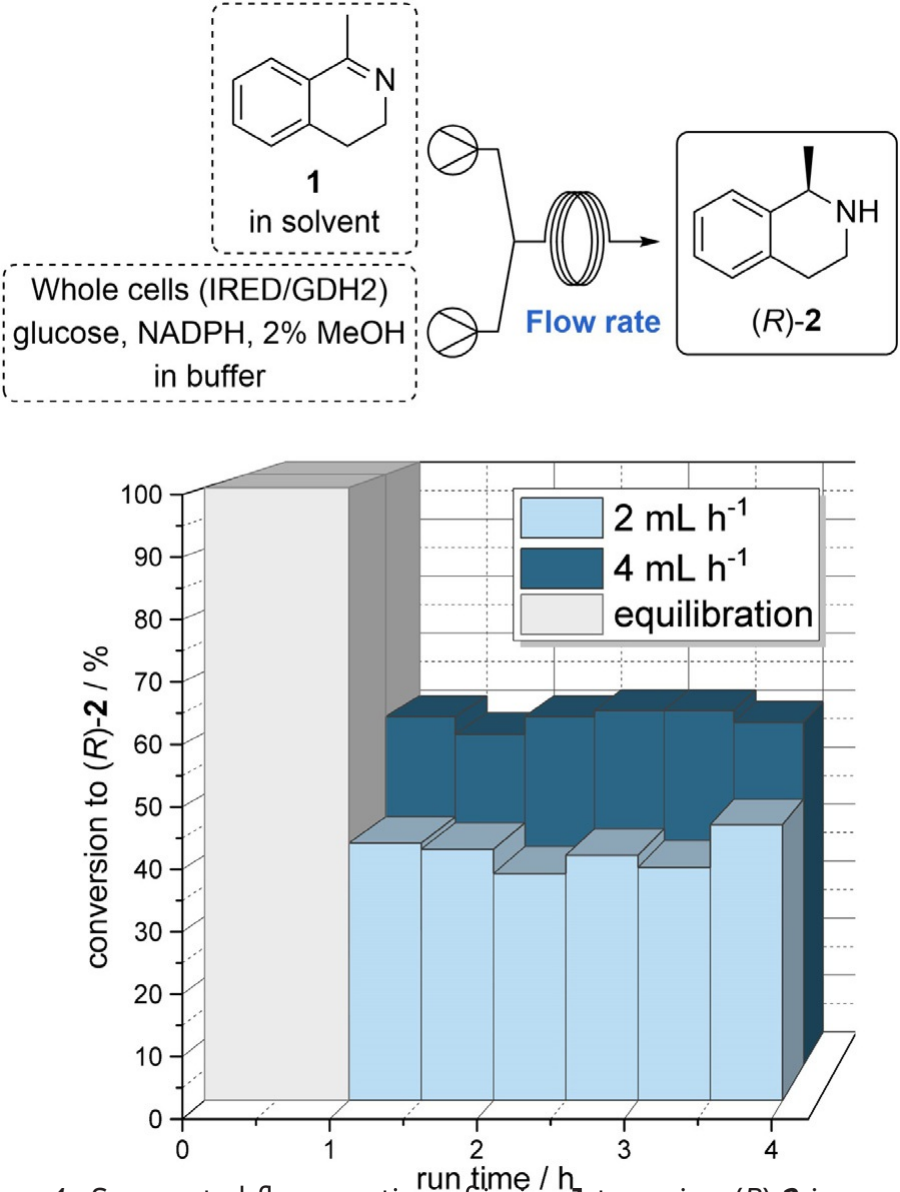
Segmented flow reaction of imine **1** to amine (*R*)‐**2** in methylcyclohexane and KP_i_ buffer solution, with an overall flow rate of 2 and 4 mL h^−1^, respectively, corresponding to 0.5 h residence time. Reaction conditions: Biphasic system with 40 mM of substrate **1** in methylcyclohexane and 10 mg mL^−1^ whole‐cell catalyst, 0.2 mM NADP^+^, 240 mM glucose, 2 % MeOH in KP_i_ buffer (50 mM, pH 7) at 30 °C and different flow rates.

With these results from the batch biotransformations in hand, we investigated the reaction in a segmented flow setup (Figure 4). We chose identical conditions to the batch experiments to ensure comparability between the batch and flow processes. Thus, an organic solution with substrate **1** (40 mM) in methylcyclohexane as well as an aqueous solution containing d‐glucose, NADP^+^, *E. coli* BL21(DE3) whole‐cells with IRED and GDH (10 mg_dcm_ mL^−1^) and methanol (2 vol%), were transferred into syringes and combined with each other through a Y‐mixer. This then resulted in a segmented flow system which was fed to a coil reactor (PFE, 0.8 mm inner diameter). It should be added that by means of the utilized (two‐channel) syringe pump, disruption of the whole‐cells caused by forces from the pump (high pressure or shear forces) is highly unlikely. The residence time was set to 0.5 h and an equilibration time of two residence times was considered to adjust a steady state. Fractions of the reaction mixture were then collected in glass vials containing a quenching solution (0.4 mL, 2 M aq. NaOH solution). The quenching method was validated in advance. The segment size is strongly dependent on the flow rate, the inner tube diameter of the reactor and the utilized mixer (Y‐ or T‐piece with 0.5 to 1 mm bore). In our experiments presented here, the segment length was found to be in a range of 0.2 to 0.8 mm.

When conducting the enzymatic reduction of **1** under these (initial) flow conditions, we were pleased to find already a significant increase in conversion compared to the batch process (Figure 4). In detail, the conversion raised up from 25 % (after 0.5 h reaction time in batch, see Figure 3) to 41 % under these non‐optimized flow conditions (with a residence time of 0.5 h, see Figure 4).

Next, we focused on optimizing the flow process. To improve mass transfer, which turned out as a crucial issue for process efficiency already in the batch mode experiments (Figure 3), we increased the flow rate and elongated the reactor length in order to intensify phase mixing without changing other process parameters. In accordance with our expectations, at an elevated flow rate of 4 mL h^−1^ the conversion could be further increased significantly, leading to the formation of amine (*R*)‐**2** with 58 % conversion (Figure 4). These findings confirm our hypothesis that mass transfer‐limited biotransformations in biphasic media can tremendously benefit from conducting them in a segmented flow mode: even compared to the analogous reaction in batch mode running at a high stirring rate of 1100 rpm, in the analogous flow process the conversion has been more than doubled (Figure 4).

Completing the investigations on the imine reduction, finally preparative experiments with product isolation were carried out in batch as well as segmented flow mode (for details, see Supporting Information). In both cases the formed product (*R*)‐**2** was isolated from reaction mixtures, which showed full conversion. In case of the batch reaction, isolation proved to be tedious due to poor phase separation. Thus, centrifugation and an additional two‐fold extraction was needed. However, even with this time‐consuming work‐up, only 75 % (>99 % purity) of the product (*R*)‐**2** could be isolated. In contrast, in case of the segmented‐flow approach, phase separation was not a critical issue and, thus, 95 % yield of the isolated product (*R*)‐**2** (95 % purity) was obtained after a simple phase separation.

### Aldoxime Dehydration in a Continuous Segmented Flow Mode: The Impact of a Surfactant on Process Efficiency

Encouraged by this positive impact of the segmented flow technology on whole‐cell processes running in biphasic media, we became interested in demonstrating the generality of this flow method by expanding it to further biocatalytic applications. In addition, we wanted to explore if whole‐cell catalysis in flow is also superior to the batch mode (and enables improved mass transfer) when surfactants are used as additives. As the model reaction for this study we chose the enzymatic key step of a recently developed cyanide‐free route for nitriles, which consists of an Oxd‐catalyzed dehydration of oximes to nitriles.[^38^, ^39^, ^40^] When focusing on aliphatic nitriles, this biocatalytic method goes beyond the common application range of enzymes in the fields of fine chemicals and pharmaceuticals, as the resulting *n*‐octanitrile (**4**) serves as a bulk chemical.^[41]^ The aldoxime dehydratase from *Bacillus* sp. OxB‐1 (OxdB), overexpressed in *E. coli* BL21(DE3),^[42]^ proved to be a suitable whole‐cell catalyst for this purpose.^[41]^

As a surfactant we used the polysorbate‐type non‐ionic surfactant Tween 20 for our studies, as this compound was successfully applied previously by Buehler et al. in another biotransformation.^[30]^ Again, we started with initial batch experiments in order to set a benchmark for the subsequent flow experiments. When conducting the Oxd‐catalyzed dehydration of *n*‐octanal oxime (**3**) in a batch mode with whole‐cells at a reaction time of 30 min, a conversion of 13 % to nitrile **4** was observed without the Tween 20 additive. In the presence of Tween 20, however, the dehydration in the presence of the Oxd‐containing whole‐cell proceeds with an improved conversion of 32 %, thus indicating the beneficial impact of a surfactant to make the water‐immiscible substrate accessible to the enzyme. Furthermore, this experiment indicates the mass transfer limitation also for this type of reaction.

**Figure 5:**
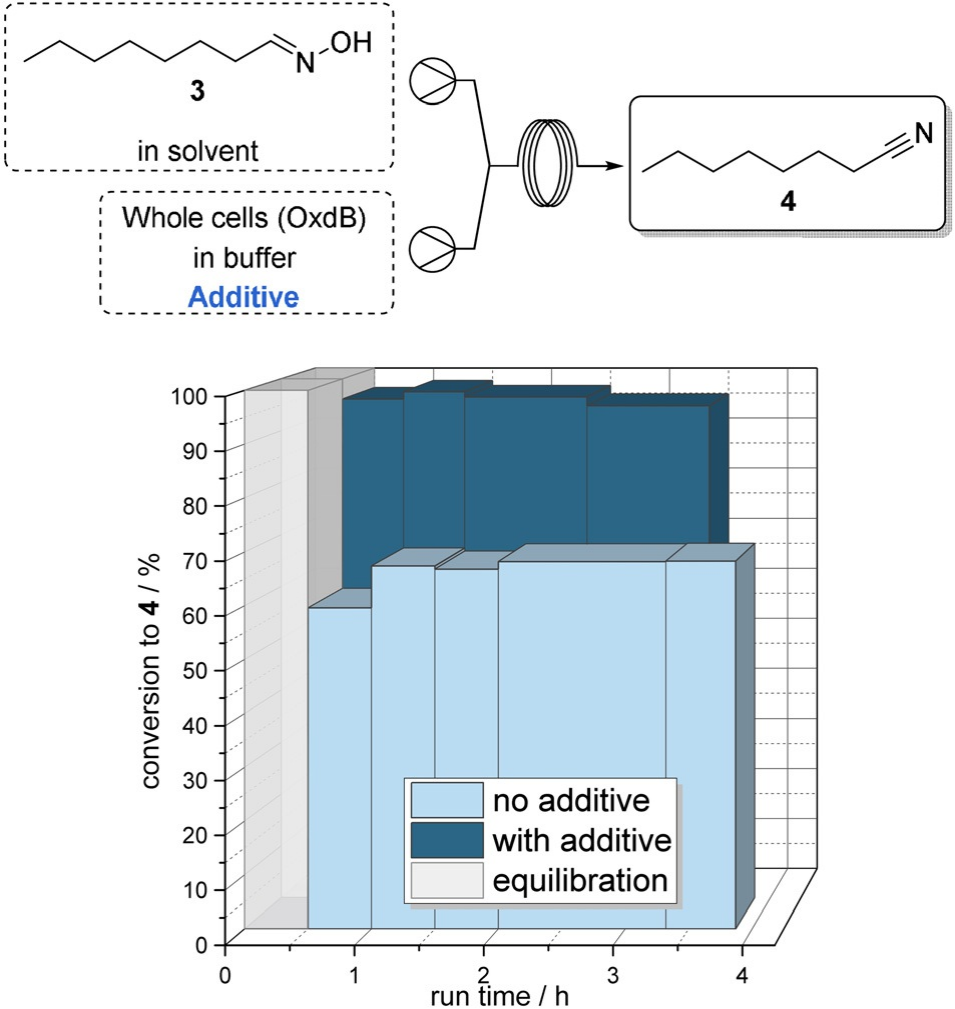
Upper part: Schematic reaction setup. Lower part: Conversion to nitrile **4** against the reactor run time (starting from switching on of the system) of a cyclohexane/buffer segmented flow approach for the OxdB‐catalysed dehydration of *n*‐octanal oxime (**3**) with and without addition of Tween 20.

When conducting the same reaction in a segmented flow mode, we found again a dramatic improvement of the conversion (Figure 5). Without the additive, an already increased average conversion to nitrile **4** of 68 % was observed, and with Tween 20 as an additive a nearly complete conversion (average of 96 %) was achieved within a short residence time of 30 min.

When switching from batch to flow mode, all other parameters were kept constant to preserve comparability between the systems (except in case of the flow process a quenching step was conducted using a 2 M aqueous HCl solution).

**Figure 6:**
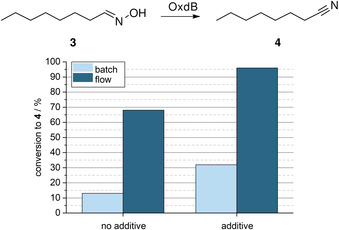
Batch versus flow approach for the OxdB‐catalysed dehydration of *n*‐octanal oxime (**3**) with and without addition of Tween 20 after 30 min reaction/ residence time.

It is noteworthy that compared to the corresponding batch experiments, the flow system performed much better (as shown in Figure 6). Thus, by means of this flow approach a space‐time yield (STY) of 12.5 g L^−1^ h^−1^ was achieved. In addition, compared to a previous result in the literature^[28]^ the biocatalyst's efficiency could be more than doubled (0.16 vs. 0.38 mg_product_ mg_wcm_^−1^).

After investigating the conversion of the reaction, we then studied the product isolation in preparative experiments. Toward this end, we compared the product isolation and resulting yields from fully converted reaction mixtures of a batch as well as a segmented flow reaction (for details, see Supporting Information). In the flow reaction, a yield of 82 % (>94 % purity) was obtained for the nitrile **4** after simple phase separation and solvent evaporation. As for the batch reaction, however, the product **4** was only obtained in a decreased yield of 64 % (>95 % purity) in spite of a more tedious work‐up consisting of centrifugation, phase separation as well as a two‐times extraction with cyclohexane (and subsequent centrifugation).

### Enzymatic Cascades in a Continuous Segmented Flow Mode: The Impact on Conversion and Product Isolation of the Emulsifying Plant Hormone Intermediate 12‐OPDA

**Scheme 2:**
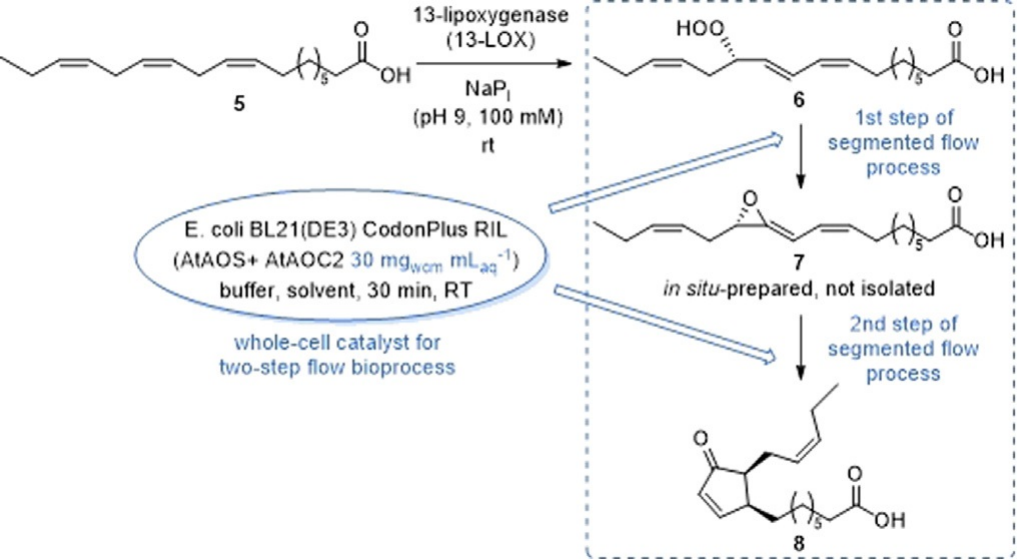
Synthesis of 12‐OPDA (**8**) starting from 13‐HPOT (**6**).

Finally, we studied the segmented flow system for the synthesis 12‐OPDA (**8**), which represents a challenging product in terms of synthetic complexity as well as product isolation. 12‐OPDA (**8**) is a prostaglandin‐related metabolite in plants^[43]^ and a precursor of jasmonic acid.^[44]^ At the same time this compound has emulsifying properties, which made product isolation in batch syntheses difficult and led to a tedious work‐up and non‐satisfactory yields. In addition, the biosynthetic steps towards this plant hormone intermediate are challenging since this reaction cascade is based on a highly labile intermediate.^[45]^ In contrast, the biosynthesis of 12‐OPDA (**8**) is much shorter than any reported chemical total syntheses,^[43]^ which makes this route attractive for synthetic purpose. We recently reported a bioprocess in batch‐mode based on this biosynthesis (Scheme 2),^[45]^ which starts with the formation of hydroperoxide **6** (HPOT) from α‐linolenic acid (**5**) by means of a 13‐lipoxygenase. Subsequently, an allene oxide synthase then catalyzes the formation of a highly labile epoxide intermediate (**7**), which then is transformed into the desired 12‐OPDA (**8**) in the presence of an allene oxide cyclase.

For the synthetic evaluation of this bioprocess in a segmented flow mode, we focused on the critical final two‐step cascade consisting of the formation of the highly labile allene oxide **7** and subsequent cyclization to 12‐OPDA (**8**). The first intermediate 13‐HPOT (**6**) was synthesized beforehand, in accordance with our previously reported protocol.^[45]^ Starting from 13‐HPOT (**6**), we conducted various batch‐type experiments, which served as a benchmark for the subsequent segmented flow reactions. In the initial batch experiments, we evaluated the influence of different buffer‐solvent systems as well as the amount of biocatalyst and additive. Details of the batch optimization are given in the Supporting Information. The reaction time was set to 30 min as for the subsequent flow process we also chose 30 min as a residence time. At this reaction time, the highest conversion to 12‐OPDA (**8**) in batch reactions was found to be 34 %. For the optimized batch‐experiment, 20 mg_wcm_ mL_aq_^−1^ of whole‐cell catalyst, 1 vol% Tween 20 and isooctane as organic solvent were used.

**Figure 7:**
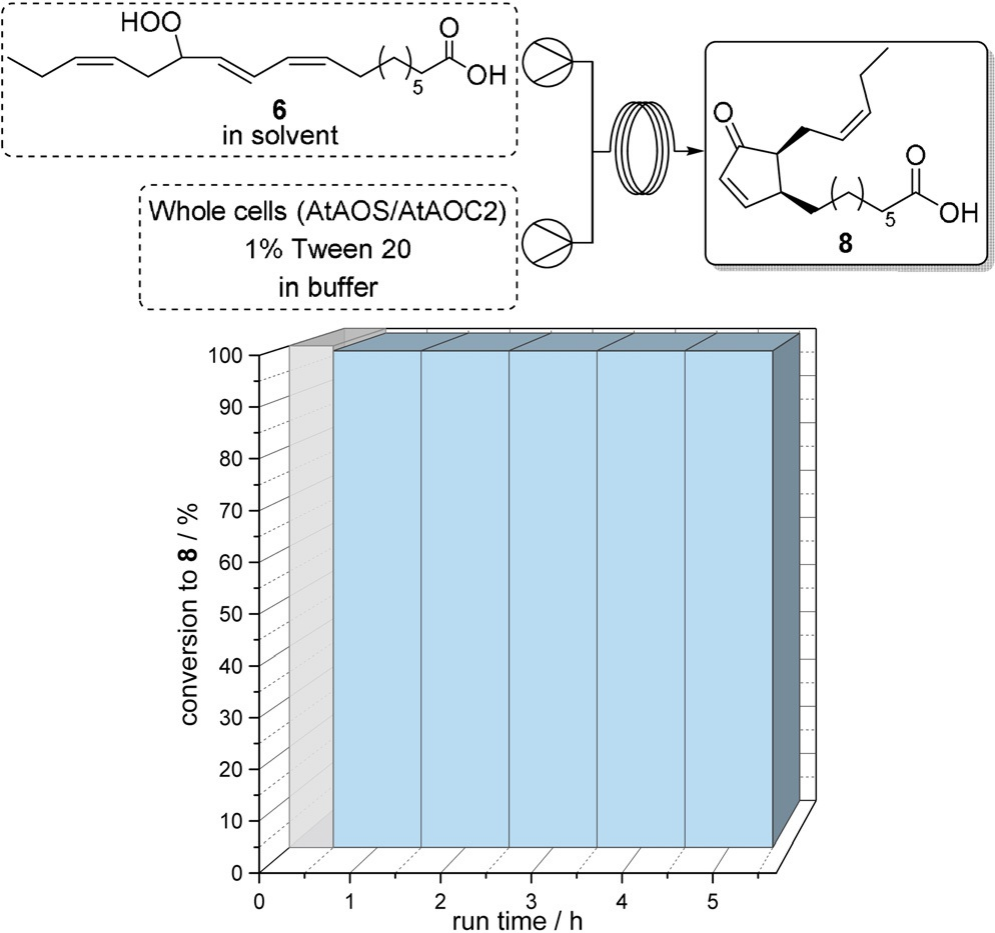
Synthesis of 12‐OPDA (**8**) in a segmented flow system with 20 mg_wcm_ mL_aq_^−1^ whole‐cells in sodium phosphate buffer (pH 8, 100 mM), 1 % Tween 20 and isooctane with 13‐HPOT (**6**) at room temperature and 30 min residence time.

For the synthesis of 12‐OPDA (**8**) in the segmented flow mode the same reaction conditions as in the batch‐reactions were used. Accordingly, the hydroperoxide substrate **6** was dissolved in isooctane and the tailor‐made whole‐cell catalyst consisting of the allene oxide synthase and allene oxide cyclase was suspended in a buffer with 1 vol% addition of Tween 20 as a surfactant. When conducting this synthesis in the segmented flow mode, we were pleased to find again a dramatic increase of the conversion, which reached in average >99 % along with a high bioprocess stability over at least a run time of 5 h (Figure 7). Thus, compared to the batch experiment (34 %) the conversion could be almost tripled when carrying out this process in a segmented flow mode while keeping all other parameters unchanged, which underlines the high efficiency of flow processes also for whole‐cell‐catalyzed transformations.

Note that utilizing whole‐cells being stored only at 4 °C and not at −20 °C is crucial for this bioprocess, as with frozen cells no conversion was observed, which can be rationalized by permeabilization of the cell membranes leading to less protection of the enzymes against the surrounding organic solvents.

**Figure 8:**
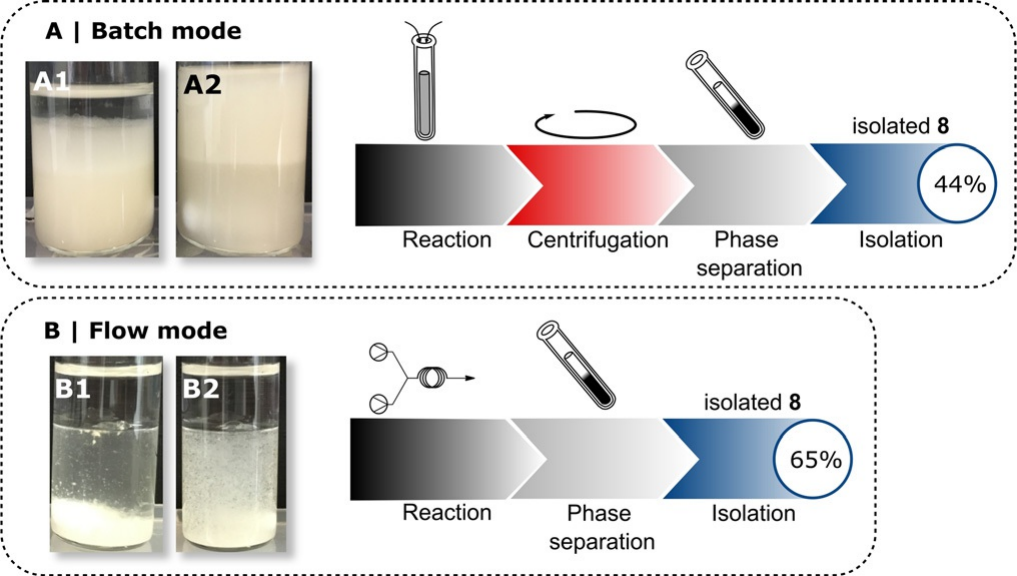
Reaction mixtures of the synthesis of 12‐OPDA (**8**) in batch mode (**A**) before quenching (left, **A1**) and after quenching (right, **A2**), as well as segmented flow mode (**B**), directly after the reaction (left, **B1**) and after sedimentation of the cells (right, **B2**). Arrow symbolises the required unit operations leading to the isolation of **8**.

Even after significantly improving the catalytic efficiency of this cascade with whole‐cells by means of a segmented flow process, product isolation as a further challenge remained. The target molecule 12‐OPDA as well as many related fatty acid‐derived compounds are known from the literature[^45^, ^46^, ^47^] to be difficult to isolate from aqueous solutions due to their emulsifying properties. Thus, we became interested in determining if the segmented flow technology is not only able to dramatically increase the catalytic efficiency, but also to simplify the downstream processing by enabling an improved phase separation. For a better comparison of the isolation efficiency of batch versus flow processes, we compared the yields of isolated 12‐OPDA (**8**) from the batch‐mode with those from the segmented flow process when starting in both cases with fully converted reaction mixtures (Figure 8). Notably, there is a clear optical difference between both reaction mixtures resulting from the biotransformations in the batch mode (Figure 8 A) and segmented flow mode (Figure 8 B).

In the batch process, a clear phase separation does not occur and an interphase is formed, which can be attributed to an emulsion containing cells and fatty acid (Figure 8, A1). Furthermore, after quenching (A2) the organic phase is nearly fully mixed with the interphase, which makes further work‐up rather difficult (Figure 8, A2). Thus, for this batch‐type bioprocess a tedious further downstream‐processing consisting of, e.g., a phase separation via centrifugation, turned out to be mandatory.

In contrast, we found that in the flow experiment a clear phase boundary was observed directly at the end of the reaction without further treatment of the reaction mixture and even without centrifugation (Figure 8, B1). Furthermore, after a short time the cells sedimented (Figure 8, B2) and the organic phase could be easily separated without further process operations. Toward this end, the organic phase was decanted, and the solvent was removed. Whereas for the batch process a yield of only 44 % could be achieved, the flow process with its more simplified work‐up led to an increased, still non‐optimized yield of 65 %. This improvement of the yield underlines the benefit of the application of whole‐cell catalysis in a segmented flow system not only for increasing the catalytic efficiency itself but also for simplifying and improving product isolation.

## Conclusion

In conclusion, we demonstrated that utilizing whole‐cell catalysts in a segmented flow mode provides an effective and at the same time easy‐to‐use methodology for enabling highly efficient biotransformations, thus overcoming existing limitations previously known from the widely used batch‐processes. In addition to up to tripled conversions to the desired products, downstream processing was found to be dramatically improved. For a selected biotransformation with known extremely challenging work‐up due to emulsification and problematic phase separation, a 1.5‐fold higher yield was obtained for the isolated product with this flow bioprocess compared to the standard batch process. Furthermore, we showed that whole‐cell catalysis can be simply implemented into a flow process even without specific prior experience or needed equipment. In addition, large‐scale production of such whole‐cell processes can potentially be easier in a flow mode (via “numbering up” of the optimized process) since these biotransformations have been shown to be very sensitive to mass transfer and stirring rate in the batch mode, which makes scale‐up challenging. A further advantage of flow processes is that shear forces can be minimized, which is beneficial for enabling a high biocatalyst stability. Moreover, constant product quality from batch to batch can also not be necessarily ensured due to the sensitivity of the system in contrast to a segmented flow system. We believe that the reported whole‐cell flow process technology has a broad reaction scope and can be applied to numerous biocatalytic systems, thus contributing to a further increase of the number of whole‐cell catalytic applications in organic synthesis.

## Conflict of interest

The authors declare no conflict of interest.

## Supporting information

As a service to our authors and readers, this journal provides supporting information supplied by the authors. Such materials are peer reviewed and may be re‐organized for online delivery, but are not copy‐edited or typeset. Technical support issues arising from supporting information (other than missing files) should be addressed to the authors.

Supplementary
