# Supplementary material for: Segmented Flow Processes to Overcome Hurdles of Whole‐Cell Biocatalysis in the Presence of Organic Solvents
Source: Angew Chem Int Ed Engl. 2021 Jun 10;60(29):15863–9. doi: 10.1002/anie.202015887 (PMC8362180; doi:10.1002/anie.202015887)
Supplement: Supplementary file 1 — Supplementary [file ANIE-60-15863-s001.pdf]

Angewandte Chemie
Eine Zeitschrift der Gesellschaft Deutscher Chemiker

Supporting Information

Segmented Flow Processes to Overcome Hurdles of Whole-Cell Biocatalysis in the Presence of Organic Solvents

*Niklas Adebar, Alina Nastke, Jana Löwe, and Harald Gröger**

anie_202015887_sm_miscellaneous_information.pdf

Supporting Information

Table of Content:

1. Standard working conditions and equipment
2. Analytics
3. Protein sequences, plasmids and expressions
    - 3.1. Imine reductase
        - 3.1.1. Preparation of *E. coli* BL21 DE(3) whole cells with IRED from *Streptomyces viridochromogenes* and GDH2 from *Bacillus subtilis*
        - 3.1.2. Gene and amino acid sequence of IRED and GDH
    - 3.2. OxdB from *Bacillus* sp. OxB-1
        - 3.2.1. Preparation of OxdB from Bacillus sp. OxB-1 whole cells
        - 3.2.2. Gene and amino acid sequences
    - 3.3. AtAOS + AtAOC2
        - 3.3.1. Preparation AOS and AOC2 from *Arabidopsis thaliana* whole cells
        - 3.3.2. Gene and amino acid sequences of AOS and AOC2
4. Synthesis
    - 4.1. Synthesis of 1-methyl-1,2,3,4-tetrahydrisoquinoline in biphasic system (solvent screening)
    - 4.2. Synthesis of 1-methyl-1,2,3,4-tetrahydrisoquinoline in biphasic system (batch mode)
    - 4.3. Synthesis of 1-methyl-1,2,3,4-tetrahydrisoquinoline in biphasic system (batch mode in preparative scale)
    - 4.4. Synthesis of 1-methyl-1,2,3,4-tetrahydrisoquinoline in biphasic system (segmented-flow mode)
    - 4.5. Synthesis of 1-methyl-1,2,3,4-tetrahydrisoquinoline in biphasic system (segmented-flow mode in preparative scale)
    - 4.6. Synthesis of octane nitrile in biphasic systems (batch mode)
    - 4.7. Synthesis and isolation of octane nitrile in biphasic systems (batch mode)
    - 4.8. Synthesis of octane nitrile in liquid/liquid segmented flow
    - 4.9. Synthesis and isolation of octane nitrile in liquid/liquid segmented flow
    - 4.10. Synthesis of 13-HPOT
    - 4.11. Synthesis of 12-OPDA in batch (analytical scale)
    - 4.12. Synthesis of 12-OPDA in batch (preparative scale)
    - 4.13. Synthesis of 12-OPDA in segmented flow (analytical scale)
    - 4.14. Synthesis of 12-OPDA in segmented flow (preparative scale)
5. ^{1}H NMR Chromatograms
6. References

1. Standard working conditions and equipment

Unless stated otherwise, reagents were obtained commercially and used without further purification. Solvent was removed under reduced pressure at 40 °C using a standard rotary evaporator, unless noted otherwise. PFE tubing, fittings, connectors and fittings were obtained from TECHLAB. A Fusion 200 touch or Fusion 4000 syringe pump by Chemyx was used along with glass syringes (S.G.E. gas tight).

2. Analytics

^1H NMR spectra were recorded on a Bruker Avance III 500 spectrometer (500 MHz) in chloroform-d3, referenced internally to the residual solvent peaks ppm (chloroform-d3: 7.26 ppm (s)) and analysed using MestReNova. Chemical shifts δ are reported in ppm to the nearest 0.01 ppm. The multiplicity of ^1H signals are indicated as following: s = singlet, d = doublet, dd = doublet of doublet, t = triplet, q = quadruplet, m = multiplet, br. = broad, or combinations thereof. Coupling constants (*J*) are reported in Hz to the nearest 0.1 Hz.

GC analysis was performed on a Shimadzu GC-2010 chromatograph equipped with an AOC-20i autoinjector, a flame ionisation detector (FID) and analysed using GCsolution software by Shimadzu. Quantification was realised by calibration measurements of the target analytes (usually six individually prepared samples distributed across an area of concentration expected for analysis of the reaction mixture). N_2 was used as carrier gas.

HPLC analysis was performed on a LC200 SFC-HPLC system of Jasco (pump PU-2080Plus, automated back-pressure regulator BP-2080Plus, column thermostat CO-2060Plus, multi-wavelength detector MD-2010Plus and autosampler AS-2059Plus). For substance separation a chiral HPLC-column Chiralpak® of Daicel was used. Analysis of the chromatograms was done by using the software Galaxy Chromatography Data System was used.

*Separation of 1-methyl-3,4-dihydroisoquinoline **1** and (R)-1-methyl-1,2,3,4-tetrahydroisoquinoline **2***

Solvents: Supercritical CO_2:EtOH (+ 0.1% diethylamine), solvent ratio: 80:20, flow rate: 1 mL min^{-1}, column temperature: 20 °C, pressure: 12 mPa, detection wavelength: 213 nm.
Conversion to amine 2 were determined by integration of UV-signals at 213 nm (UV-absorption of amine **2** had to be multiplied with a correction factor of 2.34 regarding the absorption of imine **1**.
Retention times: Amine (**2**) 15.2 min, Imine (**1**) 11.7 min.

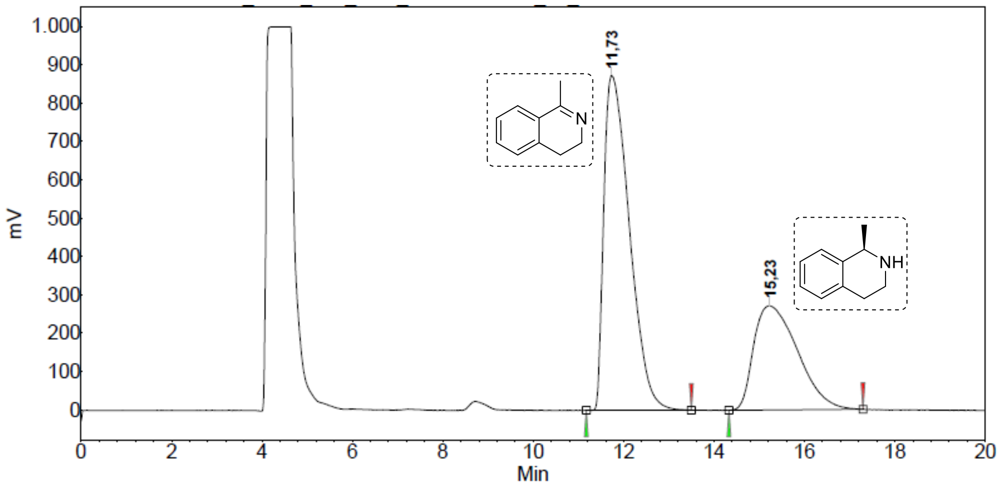

Figure S1: Exemplary SCF-HPLC spectrum of a reaction mixture separating amine **2** and imine **1**.

*Separation of octanal oxime **3** and octanenitrile **4***

Column: chiral BGB-174 (0.25 mm ID, 0.25 µm film, 30 m length) by BGB Analytik AG.
Column oven temperature program: 140 °C for 1 min, 140 to 190 °C with 20 °C min^{-1}, 190 °C for 1 min, 190 to 200 °C in 50 °C min^{-1}.
Settings: SPL1: 220 °C, pressure: 161.7 kPa, total flow: 26.2 mL min^{-1}, column flow: 2.11 mL min^{-1}, linear velocity: 46.9 cm s^{-1}, purge flow: 3.0 mL min^{-1}, split ratio: 10.0, FID: 220 °C.
Retention times: Octanenitrile (**3**) 2.4 min, octanal oxime (**4**) 2.7 min.

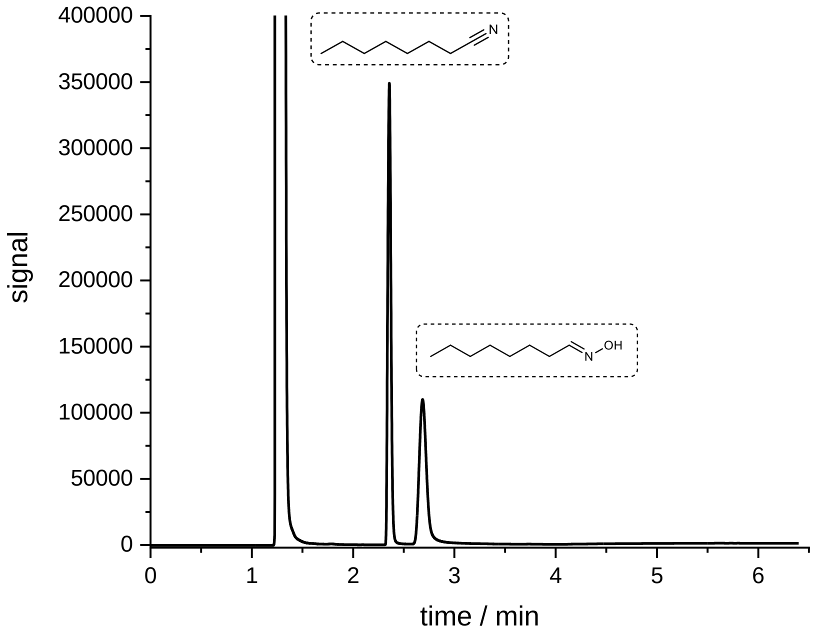

Figure S2: Exemplary GC spectrum of a reaction mixture separating nitrile **4** and oxime **3**.

3. Protein sequences, plasmids and expressions

3.1 Imine reductase

3.1.1 Preparation of *E. coli* BL21 DE(3) whole cells with IRED from *Streptomyces viridochromogenes* and GDH2 from *Bacillus subtilis*

The preparation of the *E. coli* whole-cell catalyst is following the experimental procedure of Zumbrägel *et. al*.[1] An *E. coli* strain BL21(DE3), which was used for expression, and pACYCDuet-1 vector were purchased from Novagen (Madison, USA). The whole-cell catalyst was constructed as a two-plasmid-system, harbouring the gene for the glucose dehydrogenase from *Bacillus subtilis* in a pACYCDuet-1 vector[2] and the gene for imine reductase from *Streptomyces viridochromogenes* in a pET-22b(+) vector[3]. A starting culture of *E. coli* BL21(DE3) carrying the two recombinant plasmids was cultivated over night at 37 °C in 10 mL LB-medium, containing 80 µg mL^{-1} of carbenicillin and 80 µg mL^{-1} of chloramphenicol. The main culture was incubated in 300 mL autoinduction medium (TB-medium with 2 g L^{-1} lactose and 0.5 g L^{-1} glucose) containing 100 µg mL^{-1} of carbenicillin and 100 µg mL^{-1} of chloramphenicol for 2 h at 37 °C and afterwards for 70 h at 15 °C.
After harvesting, the cells were suspended in water, resulting in a 50% suspension. The cells were then lyophilized and stored in a freezer at -20 °C.

3.1.2 Gene and amino acid sequence of IRED and GDH

IRED from *Streptomyces viridochromogenes* (N-terminal His6-Tag) 942 bp

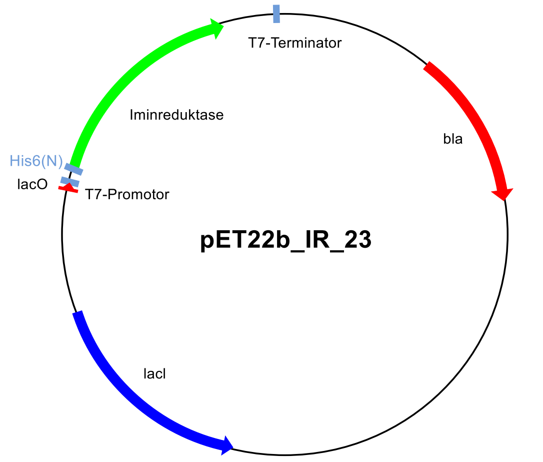

Base sequence (942 bp):
ATGCATCACCACCACCACCACAATCGCCAGTTTACCAGCACCCGCCTGAACGCAATGACGGATAATGCCTCAAGCCCGACCCCGGTTACC
CTGCTGGGCACCGGTGCGATGGGCAGCGCACTGGCTCTGCCTGGCTGGCCGCAGGTCATCCGGTTACCGTTTGGAATCGTACGCCGGCA
CGTGCAGAAGCTCTGGCCGGTGAAGGTGCAGCTGTTGCCGCAAGCGCCGATGCAGCTGTTGCCGCCAATCGCCTGTGGTTGCCTGCCTG
CTGGATGACGATTCTCTGGGTGAAGCACTGGCAACCGCTGACCTGGGCGGTCGTGATCTGGTGAACCTGACCACCGGTACGCCGGGTCA
GGGTCGTGCCCGTGCAGCTTGGGCGAAGCCCGCGGTGCCCGTTTTGTTGATGGCGGTATTATGGCAGTCCCGCCGATGATCGGCAGTCC
GGATTCCGGTGCCTTTGTGTTCTATAGCGGCTCTGCGGCCCTGTTTGAAGAACACCGTGATGGCTGGCCGTTCCGGCAGGTACCGCTTAT
GTCGGCGCAGATGCTGGTTTCGCAGCTCTGCATGACGTTGCGCTGCTGTCCGCCATGTACGGCATGTTTGGCGGTATTGCGCACGCCTTC
GCACTGATTGTCGCGAAGACATCGCACCGACCGATTTTGCGCCGCTGCTGGTGTCTTGGCTGACCGCTATGGCCCCGGCCGCACTGGAAT
CAGCCGGTAAACTGGAATCGGGTGACTATACCCGCGATGTCGTGTCAAATCTGGCAATGCAGGTTGCTGGCATCCCGACCTTCCTGCGTA
CGGCCGACGAACAAGGTGTCCGCCGGATCTGGTGCGTCCGTACCTGGACCTGATGCGTCGCCGTCTGGAATGTGGTCCGCACGCTGACG
AAGATACGACGGGTGTTATTGACCTGCTGACGGCAAGCTAA

Amino acid sequence (313):
MHHHHHHNRQFTSTRLNAMTDNASSPTPVTLLGTGAMGSALARAWLAAGHPVTVWNRTPARAEALAGEGAAVAASADAAVAANRLVVA
CLLDDDSLGEALATADLGGRDLVNLTTGTPGQGRARAAWEARGARFVDGGIMAVPPMIGSPDSGAFVFYSGSAALFEEHRDVLAVPAGTAY
VGADAGFAALHDVALLSAMYGMFGGIAHAFALIRREDIAPTDFAPLLVSWLTAMAPAALESAGKLESGDYTRDVVNLAMQVAGIPTFLRTAD
EQGVRPDLVRPYLDLMRRRLECGPHADEDTTGVIDLLTAS

GDH from *Bacillus subtilis 168* (ATCC 23857) with heatmutation E170K and Q252L on pACYCDuet-1

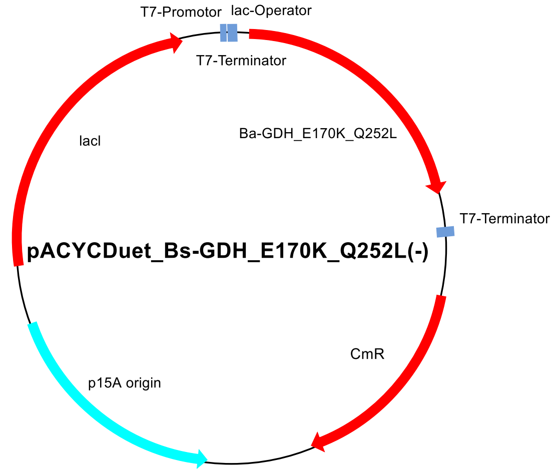

Base sequence (786 bp):
ATGTATCCGGATTTAAAAGGAAAAGTCGTCGCTATTACAGGAGCTGCTTCAGGGCTCGGAAAGGCGATGGCCATTCGCTTCGGCAAGGA
GCAGGCAAAAGTGGTTATCAACTATTATAGTAATAAACAGATCCGAACGAGGTAAAAGAAGAGGTCATCAAGGCGGGCGGTGAAGCTG
TTGTCGTCCAAGGAGATGTCACGAAAGAGGAAGATGTAAAAAATATCGTGCAAACGGCAATTAAGGAGTTCGGCACATCGATATTATGA
TTAATAATGCCGGTCTTGAAAATCCTGTGCCATCTCACGAAATGCCGCTCAAGGATTGGGATAAAGTCATCGGCACGAACTTAACGGGTG
CCTTTTTAGGAAGCCGTGAAGCGATAAATATTTCGTAGAAAACGATATCAAGGGAAATGTCATTAACATGTCCAGTGTGCACGAAGTGAT
TCCTTGGCCGTTATTTGTCCACTATGCGGCAAGTAAAGGCGGGATAAAGCTGATGACAAAGAATTAGCGTTGGAATACGCGCCGAAGGG
CATTCGCGTCAATAATATTGGGCCAGGTGCGATCAACACGCCAATCAATGCTGAAAAATTCGCTGACCCTAAACAGAAAGCTGATGTAGA
AAGCATGATTCAATGGGATATATCGGCGAACCGGAGGAGATCGCCGCAGTAGCAGCCTGGCTTGCTTCGAAGGAAGCCAGCTACGTCA
CAGGCATCACGTTATTCGCGGACGGCGGTATGACACTCTATCCTTCATTCAGGCAGGCCGCGGTTAA

Amino acid sequence (261)
MYPDLKGKVVAITGAASGLGKAMAIRFGKEQAKVVINYYSNKQDPNEVKEEVIKAGGEAVVVQGDVTKEEDVKNIVQTAIKEFGTLDIMINNA
GLENPVPSHEMPLKDWDKVIGTNLTGAFLGSREAKYFVENDIKGNVINMSSVHEVIPWPLFVHYAASKGGIKLMTKTLALEYAPKGIRVNNIGP
GAINTPINAEKFADPKQKADVESMIPMGYIGEPEEIAAVAAWLASKEASYVTGITLFADGGMTLYPSQAGRG

3.2 OxdB from *Bacillus* sp. OxB-1

3.2.1 Preparation of OxdB from *Bacillus* sp. OxB-1 whole cells

The gene for the aldoxime dehydratase from *Bacillus* sp. OxB-1 (OxdB) was cloned into a pUC18 vector using HindIII and PstI restriction sites. Whole cells containing OxdB from *Bacillus* sp. OxB-1 were prepared according to literature [4] and stored as whole cell suspension at 4 °C.

3.2.2 Gene and amino acid sequences

Oxd from *Bacillus* sp. OxB-1 (OxdB) without Tag (Accession number: GenBank: AP013294.1) Base sequence (codon-optimized for E. coli):

ATGAAAAATATGCCGGAAAATCACAATCCACAAGCGAATGCCTGGACTGCCGAATTTCCTCCTGAAATGAGCTATGTAGTATTTGCGCAG
ATTGGGATTCAAAGCAAGTCTTTGGATCACGCAGCGGAACATTTGGGAATGATGAAAAAGAGTTTCGATTTGCGGACAGGCCCCAAACA
TGTGGATCGAGCCTTGCATCAAGGAGCCGATGGATACCAAGATTCCATCTTTTTAGCCTACTGGGATGAGCCTGAAACATTTAAATCATG
GGTTGCGGATCCTGAAGTACAAAAGTGGTGGTCGGGTAAAAAAATCGATGAAAATAGTCCAATCGGGTATTGGAGTGAGGTAACGACC
ATTCCGATTGATCACTTTGAGACTCTTCATTCCGGAGAAAATTACGATAATGGGGTTTCACACTTTGTACCGATCAAGCATACAGAAGTCC
ATGAATATTGGGGAGCAATGCGCGACCGCATGCCGGTGTCTGCCAGTAGTGATTTGGAAAGCCCCCTTGGCCTTCAATTACCGGAACCC
ATTGTCCGGGAGTCTTTCGGAAAACGGCTAAAAGTCACGGCGCCGGATAATATTTGCTTGATTCGAACCGCTCAAAATTGGTCTAAATGT
GGTAGCGGGGAAAGGGAAACGTATATAGGACTAGTGGAACCGACCCTCATAAAAGCGAATACGTTTCTTCGTGAAAATGCTAGTGAAA
CAGGCTGTATTAGTTCAAAATTAGTCTATGAACAGACCCATGACGGCGAAATAGTAGATAAATCATGTGTCATCGGATATTATCTCTCCAT
GGGGCATCTTGAACGCTGGACGCATGATCATCCAACACATAAAGCGATCTACGGAACCTTTTATGAGATGTTGAAAAGGCATGATTTTAA
GACCGAACTTGCTTTATGGCACGAGGTTTCGGTGCTTCAATCCAAAGATATCGAGCTTATCTATGTCAACTGCCATCCGAGTACTGGATTT
CTTCCATTCTTTGAAGTGACAGAAATTCAAGAGCCTTTACTGAAAAGCCCTAGCGTCAGGATCCAGTGA

Amino acid sequence:

MKNMPENHNPQANAWTAEFPPEMSYVVFAQIGIQSKSLDHAAEHLGMMKKSFDLRTGPKHVDRALHQGADGYQDSIFLAYWDEPETFKS
WVADPEVQKWWSGKKIDENSPIGYWSEVTTIPIDHFETLHSGENYDNGVSHFVPIKHTEVHEYWGAMRDRMPVSASSDLESPLGLQLPEPIV
RESFGKRLKVTAPDNICLIRTAQNWSKCGSGERETYIGLVEPTLIKANTFLRENASETGCISSKLVYEQTHDGEIVDKSCVIGYYLSMGHLERWTH
DHPTHKAIYGTFYEMLKRHDFKTELALW HEVSVLQSKDIELIYVNCHPSTGFLPFFEVTEIQEPLLKSPSVRIQ

3.3 AtAOS + AtAOC2

3.3.1 Preparation AOS and AOC2 from *Arabidopsis thaliana whole cells*

The gene for the AOS from *Arabidopsis thaliana* (AtAOS) was cloned into a pET28a(+) vector using XhoI and NcoI restriction sites. The gene for the AOC2 from *Arabidopsis thaliana* (AtAOC) was cloned into a pQE30 vector using BamHI and SalI restriction sites. Afterwards, plasmid-DNA was added to chemical competent cells (50 µL) and incubated for 30 minutes on ice. The cells were heated at 42 °C for 90 seconds and incubated again for five minutes on ice. Afterwards 1 mL of LB media was added. The mixture was heated for three hours at 37°C and 800 rpm. Subsequently, the cells were cultured on LB agar plates with suitable antibiotic and incubated overnight at 37 °C. A colony was isolated and transferred into an autoclaved 100 mL flask with 20 mL LB-medium and 20 µL of antibiotica. The mixture was incubated over night at 37 °C. TB-medium (200 mL) with 200 µL of antibiotic were transferred in autoclavated flasks. The medium was inoculated with 1% overnight culture. The

cultures were grown at 37 °C and 180 rpm. When the culture reached an OD of 0.6-0.8, cell cultures were induced with 200 µL of IPTG (1M). Afterwards cells were harvested (4000x g, 4 °C, 30 minutes) and stored at 4 °C.

3.3.2 Gene and amino acid sequences of AOS and AOC2

Allene Oxide Synthase (AOS) from *Arabidopsis thaliana* in pET28(a)+ Nucleotide-sequence (Accession number: GenBank: AB007647), without chloroplast target sequence[5] and solubility-sequence GCAAAAAAAACATCATCA[6] and C-terminal His6 tag.

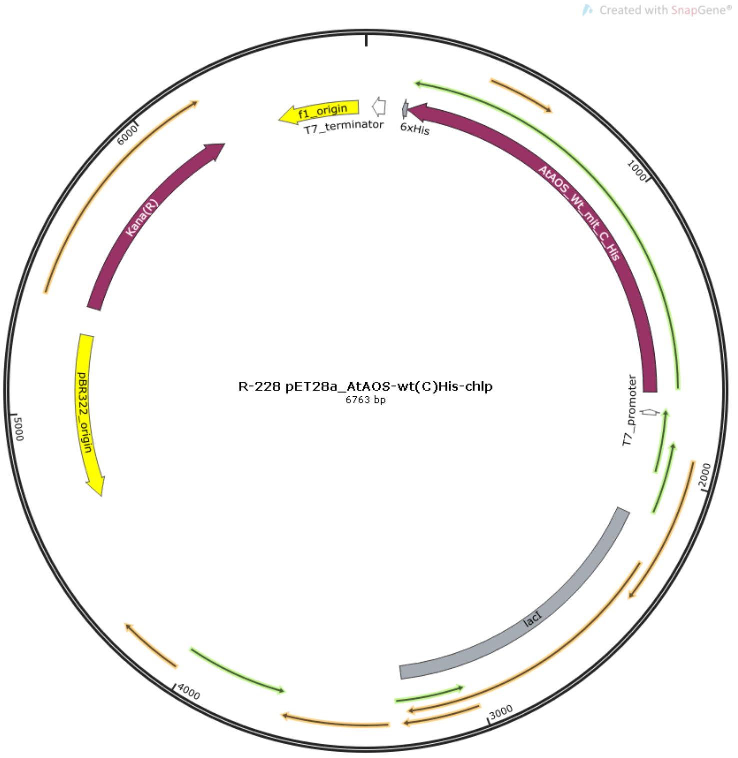

ATGGCAAAAAAAACATCATCAGCATCAGGATCAGAAACACCAGACCTAACAGTAGCGACACGAACCGGATCCAAAGATCTCCCGATCCG
AAACATACCGGGAAACTACGGTTTACCAATCGTAGGACCAATCAAAGACCGTTGGGATTACTTTTACGACCAAGGAGCTGAAGAGTTCTT
CAAATCACGAATCCGTAAATACAACTCCACGGTGTACAGAGTCAACATGCCACCGGGAGCTTTTATCGCCGAGAATCCACAAGTCGTGGC
TTTACTCGACGGTAAAAGCTTCCCGGTTTTATTCGATGTCGATAAAGTCGAAAAGAAAGATCTTTTCACCGGTACTTACATGCCGTCAACG
GAACTAACCGGAGGCTACCGTATCCTCTCGTACCTCGATCCATCGGAGCCTAAACACGAAAAGCTCAAAAATCTCCTTTTCTTCCTCCTCA
AGTCATCTCGAAACCGGATCTTCCCTGAGTTTCAAGCTACTTACTCCGAGCTTTTCGATTCTTTGGAGAAAGAGCTTTCCCTTAAAGGGAA
AGCGGATTTCGGCGGTTCCAGCGACGGAACCGCCTTTAATTTCTTGGCTCGGGCTTTCTACGGGACGAATCCCGCAGATACAAAGCTCAA
AGCCGACGCTCCGGGTTTGATCACTAAATGGGTTTTATTCAATCTCCATCCATTACTCTCTATTGGTTTACCGAGAGTTATAGAAGAACCTC
TCATCCATACATTTAGTCTACCACCGGCGTTAGTCAAATCTGATTACCAGAGACTCTACGAGTTTTTCTTAGAATCCGCCGGTGAGATTCTC
GTTGAAGCCGATAAATTGGGTATCTCACGAGAAGAAGCTACTCACAATCTTCTCTTCGCCACGTGCTTCAACACGTGGGGTGGGATGAA
GATTTTGTTTCCGAATATGGTTAAACGTATCGGGCGGGCGGGTCATCAAGTTCATAACCGATTAGCGGAGGAGATTAGATCTGTGATTAA
ATCCAACGGCGGAGAACTCACGATGGGAGCGATTGAGAAAATGGAGTTAACCAAATCAGTGGTTTACGAATGTCTCCGGTTTGAACCAC
CGGTTACGGCTCAATACGGTAGAGCGAAGAAGGATCTGGTTATCGAAAGCCACGACGCGGCGTTTAAAGTCAAAGCCGGTGAAATGCT
TTACGGTTATCAACCGTTGGCGACGAGAGATCCGAAGATTTTTGATCGGGCGGATGAGTTTGTGCCGGAGAGATTCGTCGGAGAAGAA
GGAGAGAAGCTTTTGAGGCATGTGTTGTGGTCGAATGGACCGGAGACGGAGACTCCGACGGTGGGGAATAAACAATGCGCCGGTAAG
GATTTTGTTGTTTTGGTGGCGAGGTTGTTTGTGATTGAGATTTTCCGGCGATATGATTCGTTTGATATTGAGGTTGGTACGTCGCCGTTAG
GAAGCTCCGTTAATTTCTCGTCGTTAAGGAAAGCTAGCTTTGTCGACAAGCTTGCGGCCGCACTCGGGCACCACCACCACCACCACTAA

Amino acid sequence

MAKKTSSASGSETPDLTVATRTGSKDLPIRNIPGNYGLPIVGPIKDRWDYFYDQGAEEFFKSRIRKYNSTVYRVNMPPGAFIAENPQVVALLDGK
SFPVLFDVDKVEKKDLFTGTYMPSTELTGGYRILSYLDPSEPKHEKLKNLLFFLLKSSRNRIFPEFQATYSELFDSLEKELSLKGKADFGGSSDGTAF
NFLARAFYGTNPADTKLKADAPGLITKWVLFNLHPLLSIGLPRVIEEPLIHTFSLPPALVKSDYQRLYEFFLESAGEILVEADKLGISREEATHNLLFA
TCFNTWGGMKILFPNMVKRIGRAGHQVHNRLAEEIRSVIKSNGGELTMGAIEKMELTKSVVYECLRFEPPVTAQYGRAKKDLVIESHDAAFKV
KAGEMLYGYQPLATRDPKIFDRADEFVPERFVGEEGEKLLRHVLWSNGPETETPTVGNKQCAGKDFVVLVARLFVIEIFRRYDSFDIEVGTSPLG
SSVNFSSLRKASFVDKLAAALGHHHHHH

Allene Oxide Cyclase (AOC2) from *Arabidopsis thaliana* in pQE30 wild-type sequence, without chloroplast target sequence[5] and N-terminal His6 tag

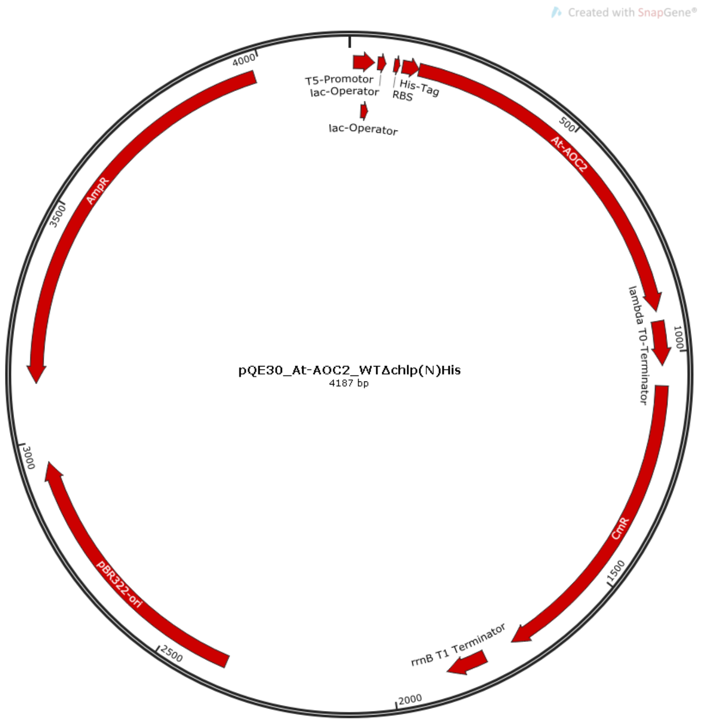

ATGAGAGGATCGCATCACCATCACCATCACGGATCCATGCTTGGTTCCTCTAAATCCTTCCAAAATCTTGGTATCTCATCTAACGGTTCAG
ATTTCTCCTATCCATCAAGTTTCACTGCCAAGAAGAACCTCACTGCTTCTCGAGCTCTCTCCCAAAACGGGAATATCGAAAACCCTAGACC
AAGCAAAGTTCAAGAACTGAGTGTGTACGAAATCAACGAATTAGATCGACACAGCCCCAAGATTCTTAAAAACGCATTCAGCTTAATGTT
CGGTCTCGGAGATCTCGTACCATTCACAAACAAACTCTACACAGGCGATCTCAAGAAACGCGTGGGCATCACGGCAGGTCTCTGCGTCGT
CATCGAACACGTCCCAGAGAAGAAAGGTGAAAGATTCGAAGCTACTTATAGCTTCTACTTCGGAGACTATGGCCACTTGTCCGTTCAAGG
ACCATACTTGACTTACGAGGATTCGTTCCTCGCCATCACTGGTGGTGCTGGAATCTTTGAAGGTGCCTACGGACAGGTCAAGCTTCAACA
GCTTGTGTATCCGACAAAACTGTTCTACACTTTTTACCTTAAAGGGTTGGCTAATGATTTGCCGTTGGAGCTCACCGGAACACCGGTACCG
CCGTCTAAGGACATAGAGCCGGCGCCGGAAGCTAAGGCACTGGAGCCTAGCGGAGTTATAAGTAACTATACCAACTAA

Amino acid-sequence

MRGSHHHHHHGSMLGSSKSFQNLGISSNGSDFSYPSSFTAKKNLTASRALSQNGNIENPRPSKVQELSVYEINELDRHSPKILKNAFSLMFGLG
DLVPFTNKLYTGDLKKRVGITAGLCVVIEHVPEKKGERFEATYSFYFGDYGHLSVQGPYLTYEDSFLAITGGAGIFEGAYGQVKLQQLVYPTKLFYT
FYLKGLANDLPLELTGTPVPPSKDIEPAPEAKALEPSGVISNYTN

4 Synthesis

4.1 Synthesis of 1-methyl-1,2,3,4-tetrahydrisoquinoline in biphasic system (solvent screening)

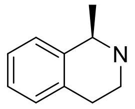

2, $C_{10}H_{12}N$
146,21 g·mol^{-1}

In a 1.5 mL-Eppendorf vessel 125 µL of 4 mg_{dcm} mL^{-1} (in KP_i-buffer 50 mM, pH 7) of whole cells containing IRED and GDH (c_{end} = 2 mg_{dcm} mL^{-1}), 25 µL of 2 mM NADP-solution$_{(aq.)}$ (0.05 µmol, 0.2 mM), 60 µL of 1 M *D*-glucose-solution$_{(KPi,\ 50\ mM)}$ (0.06 mmol, 240 mM) and methanol (10 µL, 2 %) were added and filled up with KP_i -buffer (50 mM, pH 7) to a total volume of 250 µL. Another 250 µL of 40 mM 1-methy-3,4-dihydroisoquinoline **1** (0.01 mmol) in organic solvent was added and the reaction mixture was shaken in a thermoshaker at 30 °C for 6 h (850 rpm). The reaction was analysed using SFC-HPLC by taking a 50 µL-sample of the organic phase and diluting it with ethyl acetate (1:4).

SFC-HPLC: Amine (**2**) 15.2 min, Imine (**1**) 11.7 min.

^{1}H NMR (500 MHz, Chloroform-*d*) δ / ppm = 7.14 (q, *J* = 6.5 Hz, 3H), 7.08 (d, *J* = 7.0 Hz, 1H), 4.13 (q, *J* = 6.6 Hz, 1H), 3.28 (dt, *J* = 11.5, 4.9 Hz, 1H), 3.04 (td, *J* = 12.9, 10.9, 4.8 Hz, 1H), 2.89 (dt, *J* = 14.5, 6.9 Hz, 1H), 2.76 (dt, *J* = 17.1, 4.5 Hz, 1H), 2.33 (s, 2H), 1.48 (d, *J* = 6.6 Hz, 3H).
^{13}C NMR (126 MHz, $CDCl_3$) δ / ppm = 140.29, 134.72, 129.34, 126.17, 126.08, 126.05, 77.41, 77.16, 76.91, 51.63, 41.71, 29.94, 22.69.

Table S1: Results of the IRED-catalysed reduction of 1-methy-3,4-dihydroisoquinoline **1** in two-phased organic solvent/buffer batch system. Results shown in percentage appearing in crude reaction mixture analysed by SFC-HPLC.

Org. Solvent	*Conversion to amine **2** / %*
Cyclohexane	6
Ethyl acetate	0
Isooctane	8
Methylcyclohexane	10
Methyltetrahydrofurane	0
MTBE	0

4.2 Synthesis of 1-methyl-1,2,3,4-tetrahydrisoquinoline in biphasic system (batch mode)

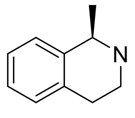

2, $C_{10}H_{12}N$
146.21 g·mol^{-1}

In a glass vial, 1 mL of 4 mg_{dcm} mL^{-1} respectively 20 mg_{dcm} mL^{-1} (in KP_i-buffer 50 mM, pH 7) of whole cells containing IRED and GDH (c_{end} = 2 mg_{dcm} mL^{-1} / 20 mg_{dcm} mL^{-1}), 200 µL of 2 mM NADP-solution$_{(aq.)}$ (0.4 µmol, 0.2 mM), 480 µL of 1 M *D*-glucose-solution$_{(KPi)}$ (0.48 mmol, 240 mM) and methanol (40 µL, 2 %) were added and filled up with KP_i -buffer (50 mM, pH 7) to a total volume of 2 mL. Another 2 mL of 40 mM 1-methyl-3,4-dihydroisoquinoline **1** (0.08 mmol) in methylcyclohexane were added and the reaction mixture was stirred with a magnetic stirring bar at 30 °C (500 rpm / 1100 rpm). The reaction was monitored using SFC-SFC-HPLC by taking 100 µL-samples of organic phase and diluting it with ethyl acetate (1:4). In case of mixed phases, 100 µL-samples were taken and were quenched with 50 µL of 2 M NaOH-solution $_{(aq.)}$. Samples were then extracted by vigorous shaking with 150 µL ethyl acetate and subsequent centrifugation (2 min, 17000 rpm) was performed to obtain a clear phase separation.

SFC-HPLC: Amine (**2**) 15.2 min, Imine (**1**) 11.7 min.

^{1}H NMR (500 MHz, Chloroform-*d*) δ / ppm = 7.14 (q, *J* = 6.5 Hz, 3H), 7.08 (d, *J* = 7.0 Hz, 1H), 4.13 (q, *J* = 6.6 Hz, 1H), 3.28 (dt, *J* = 11.5, 4.9 Hz, 1H), 3.04 (td, *J* = 12.9, 10.9, 4.8 Hz, 1H), 2.89 (dt, *J* = 14.5, 6.9 Hz, 1H), 2.76 (dt, *J* = 17.1, 4.5 Hz, 1H), 2.33 (s, 2H), 1.48 (d, *J* = 6.6 Hz, 3H).
^{13}C NMR (126 MHz, $CDCl_3$) δ / ppm = 140.29, 134.72, 129.34, 126.17, 126.08, 126.05, 77.41, 77.16, 76.91, 51.63, 41.71, 29.94, 22.69.

Table S2: Results of the IRED-catalysed reduction of 1-methyl-3,4-dihydroisoquinoline **1** in a two-phased (methyl cyclohexane/KP_i-buffer) batch system with a whole-cell concentration of 2 mg_{dcm} mL^{-1}, stirring at 30 °C and 1100 rpm (mixed phases). Results shown in percentage appearing in crude reaction mixture analysed by SFC-HPLC.

entry	*reaction time*	*Conversion to amine **2** / %*
1	15 min	2
2	30 min	4
3	45 min	8
4	1 h	10
5	2 h	18
6	3 h	21
7	4 h	23
8	5 h	25
9	6 h	36

Table S3: Results of the IRED-catalysed reduction of 1-methyl-3,4-dihydroisoquinoline **1** in a two-phased (methylcyclohexane/KP_i-buffer) batch system with a whole-cell concentration of 10 mg_{dcm} mL^{-1}, stirring at 30 °C and 1100 rpm (mixed phases). Results shown in percentage appearing in crude reaction mixture analysed by SFC-HPLC.

entry	*reaction time*	*Conversion to amine **2** / %*
1	15 min	12
2	30 min	25
3	45 min	42
4	1 h	60
5	2 h	100
6	3 h	100
7	4 h	100
8	5 h	100
9	6 h	100

Table S4: Results of the IRED-catalysed reduction of 1-methyl-3,4-dihydroisoquinoline **1** in a two-phased (methylcyclohexane/KP_i-buffer) batch system with a whole-cell concentration of 10 mg mL^{-1}, stirring at 30 °C and 500 rpm (seperated phases). Results shown in percentage appearing in crude reaction mixture analysed by SFC-HPLC.

entry	*reaction time*	*Conversion to amine **2** / %*
1	15 min	0
2	30 min	5
3	45 min	7
4	1 h	11

4.3 Synthesis of 1-methyl-1,2,3,4-tetrahydrisoquinoline in biphasic system (batch mode in preparative scale)

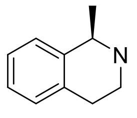

2, $C_{10}H_{12}N$
146.21 g·mol^{-1}

In a round bottom flask, 10 mL of 20 mg_{dcm} mL^{-1} (in KP_i-buffer 50 mM, pH 7) of whole cells* containing IRED and GDH (c_{end} = 10 mg_{dcm} mL^{-1}), 2 mL of 2 mM NADP-solution$_{(aq.)}$ (4 µmol, 0.2 mM), 4.8 mL of 1 M *D*-glucose-solution$_{(KPi)}$ (4.8 mmol, 240 mM) and methanol (400 µL, 2 %) were added and filled up with KP_i -buffer (50 mM, pH 7) to a total volume of 20 mL. Another 20 mL of 40 mM 1-methyl-3,4-dihydroisoquinoline **1** (116 mg, 0.8 mmol) in methylcyclohexane were added and the reaction mixture was stirred with a magnetic stirring bar at 30 °C 1100 rpm. After 1 h the reaction was quenched using 20 mL of 2 M aq. NaOH solution. For phase separation, the solution was transferred into 50 mL falcons and were centrifuged at 20 000 x g for 20 min. The organic phase was separated, while the aq. phase was extracted two more times with ethyl acetate (25 mL) and were each time centrifuged (20 000 x g, 20 min) for phase separation. The combined organic phases were dried over $MgSO_4$ and concentrated under reduced pressure, yielding the product with 75% (88 mg, 0.6 mmol, >99% purity). Analysis of the product succeeded *via* ^{1}H NMR spectroscopy.

^{1}H NMR (500 MHz, Chloroform-*d*) δ / ppm = 7.20 – 7.11 (m, 3H), 7.10 – 7.06 (m, 1H), 4.11 (q, J = 6.7 Hz, 1H), 3.27 (dt, J = 12.6, 5.0 Hz, 1H), 3.03 (td, J = 12.9, 8.8, 4.7 Hz, 1H), 2.88 (dt, J = 14.8, 8.8, 5.5 Hz, 1H), 2.74 (dt, J = 16.3, 4.7 Hz, 1H), 1.46 (d, J = 6.7 Hz, 3H).

*The activity of whole-cells used in this reaction was higher, compared to the ones used in other reactions.

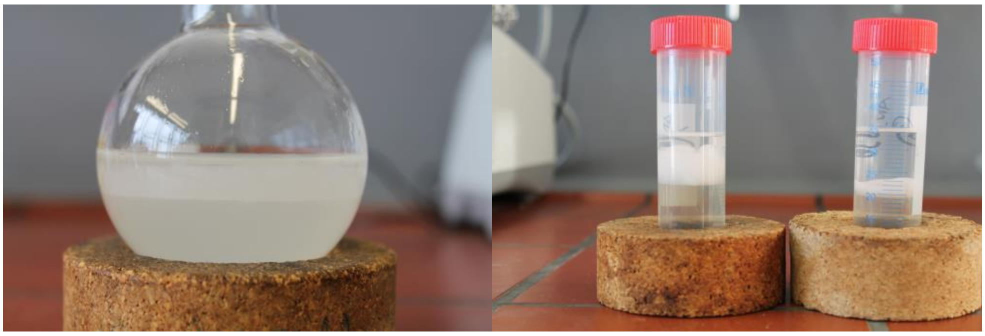

Figure S3: Left - Biphasic reaction mixture after batch reaction; Right - biphasic batch reaction mixture after extraction and centrifugation.

4.4 Synthesis of 1-methyl-1,2,3,4-tetrahydrisoquinoline in biphasic system (segmented-flow mode)

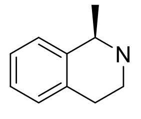

2, $C_{10}H_{12}N$
146.21 g·mol^{-1}

A syringe pump was connected to a Y-mixer (0.5 mm ID), which was connected to a tubular reactor (PFE, 0.8 mm ID, 1 mL / 2 mL). Temperature (30 °C) was controlled by a water bath. For the organic solution, 1-methyl-3,4-dihydroisoquinoline **1** (0.2 mmol, 40 mM) was dissolved in methyl cyclohexane (to 5 mL). For the aqueous solution, 2.5 mL of a 20 mg_{dcm} mL^{-1} (in KP_i-buffer 50 mM, pH 7) of whole-cells containing IRED and GDH (c_{end} = 10 mg_{dcm} mL^{-1}) were mixed with 500 µL of 2 mM NADP-solution$_{(aq.)}$ (1.0 µmol, 0.2 mM), 1.2 mL of 1 M *D*-glucose-solution$_{(KPi)}$ (1.2 mmol, 240 mM) and methanol (100 µL, 2 %) were added and filled up with KP_i-buffer (50 mM, pH 7) to a total volume of 5 mL. To start the reaction, the syringe pump was set up (flow rate: each channel 1 mL·h^{-1} / 2 mL·h^{-1}, τ = 30 min). The reaction solution was collected fractionated in glass vials containing 1 mL of 2 M NaOH-solution$_{(aq.)}$ for quenching. For SFC-HPLC analysis, 50 mL-samples of the organic phase were taken and diluted with ethyl acetate (1:4) and analysed via SFC-HPLC.

SFC-HPLC: Amine (**2**) 15.2 min, Imine (**1**) 11.7 min.

^{1}H NMR (500 MHz, Chloroform-*d*) δ / ppm = 7.14 (q, *J* = 6.5 Hz, 3H), 7.08 (d, *J* = 7.0 Hz, 1H), 4.13 (q, *J* = 6.6 Hz, 1H), 3.28 (dt, *J* = 11.5, 4.9 Hz, 1H), 3.04 (td, *J* = 12.9, 10.9, 4.8 Hz, 1H), 2.89 (dt, *J* = 14.5, 6.9 Hz, 1H), 2.76 (dt, *J* = 17.1, 4.5 Hz, 1H), 2.33 (s, 2H), 1.48 (d, *J* = 6.6 Hz, 3H).
^{13}C NMR (126 MHz, $CDCl_3$) δ / ppm = 140.29, 134.72, 129.34, 126.17, 126.08, 126.05, 77.41, 77.16, 76.91, 51.63, 41.71, 29.94, 22.69.

Table S5: Results of the IRED-catalysed reduction of 1-methyl-3,4-dihydroisoquinoline **1** in methylcyclohexane/KP_i buffer in segmented flow system with a total flow rate of 2 mL h^{-1}. Results shown in percentage appearing in crude reaction mixture analysed by SFC-HPLC.

entry	*fraction time*	*Conversion to amine **2** / %*
1	0.5 – 1 h	42
2	1 – 1.5 h	41
3	1.5 – 2.5 h	37
4	2.5 – 3.5 h	40
5	3.5 – 4.0 h	38
6	4.0 – 4.5 h	45

Table S6: Results of the IRED-catalysed reduction of 1-methyl-3,4-dihydroisoquinoline **1** in methylcyclohexane/KP_i buffer in segmented flow system with a total flow rate of 4 mL h^{-1}. Results shown in percentage appearing in crude reaction mixture analysed by SFC-HPLC.

entry	*fraction time*	*Conversion to amine **2** / %*
1	0.5 – 1 h	59
2	1 – 1.5 h	56
3	1.5 – 2 h	56
4	2 – 2.5 h	59
5	2.5 – 3 h	60
6	3 – 3.5 h	60

4.5 Synthesis of 1-methyl-1,2,3,4-tetrahydrisoquinoline in biphasic system (segmented-flow mode in preparative scale)

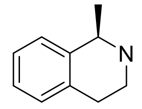

2, $C_{10}H_{12}N$
146.21 g·mol^{-1}

The segmented flow reaction in preparative scale was performed in accordance with the analytical scale reaction (Fehler! Verweisquelle konnte nicht gefunden werden.) with a longer overall run time, whereby 10 mL of 40 mM substrate solution (58 mg, 0.4 mmol) in methylcyclohexane were converted. The aqueous solution, also in accordance with the analytical flow-reaction, composed of 10 mg_{dcm} mL^{-1} (in KP_i-buffer 50 mM, pH 7) of whole-cells containing IRED* and GDH, 0.2 mM NADP, 240 mM *D*-glucose and 2% methanol in KP_i-buffer (50 mM, pH 7). The selected reactor (PFE, 0.8 mm ID, 2 mL) and flow rate (each channel 1 mL·h^{-1}) resulted in a residence time of τ = 60 min. In total, 20 mL of reaction solution was collected in a flask containing 20 mL of 2 M NaOH-solution$_{(aq.)}$ for quenching. The quenching solutions were stirred gently using a magnetic stirrer without mixing the phases. The organic phase was separated, dried over $MgSO_4$ and concentrated under reduced pressure, yielding the product with 95% (56 mg, 0.38 mmol, 95% puritiy). Analysis of the product succeeded *via* ^{1}H NMR spectroscopy.

^{1}H NMR (500 MHz, Chloroform-*d*) δ / ppm = 7.14 (q, *J* = 6.9, 3H), 7.08 (d, *J* = 7.1 Hz, 1H), 4.11 (q, *J* = 6.7 Hz, 1H), 3.27 (dt, *J* = 12.6, 5.0 Hz, 1H), 3.03 (dt, *J* = 12.8, 8.8, 4.7 Hz, 1H), 2.87 (dt, *J* = 14.9, 8.9, 5.5 Hz, 1H), 2.74 (dt, *J* = 16.5, 4.8 Hz, 1H), 1.46 (d, *J* = 6.7 Hz, 3H).

*The activity of whole-cells used in this reaction was higher, compared to the ones used in other reactions.

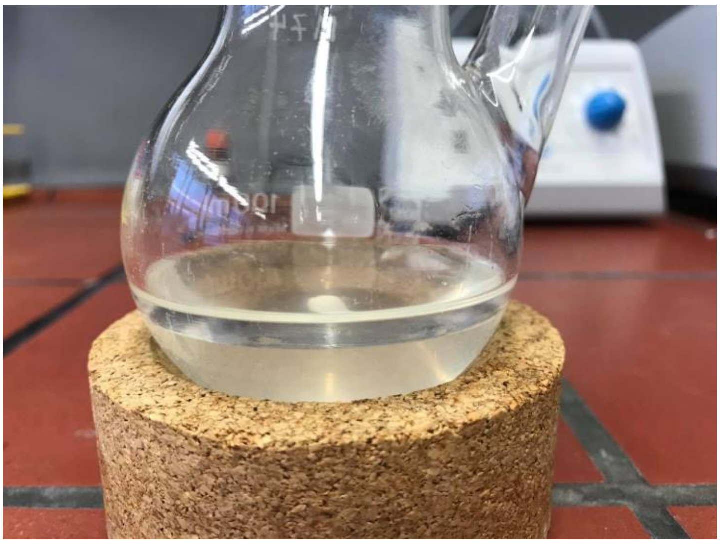

Figure S4: Biphasic reaction mixture after flow reaction.

4.6 Synthesis of octane nitrile in biphasic systems (batch mode)

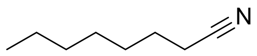

4, $C_8H_{15}N$
125.22 g·mol[-1]

In a glass vial, whole cells containing OxdB (100 µL, 333 mg mL^{-1} wet cell mass) were suspended in KP_i buffer (900 µL, 50 mM, pH 7). A solution of octanal oxime (17.3 mg, 0.1 mmol, $c_{organic}$: 100 mM) in cyclohexane (to 1 mL) was added at 30 °C. The reaction mixture was stirred for 2 h with a magnetic stirring bar (400 rpm) at 30 °C. The reaction was monitored by GC analysis of diluted organic phase (20 µL) of the reaction mixture with ethyl acetate (100 µL).

GC: Octane nitrile (**4**) 2.4 min, octanal oxime (**3**) 2.7 min.

Table S7: Results of the OxdB-catalysed dehydration of octanal oxime (**3**) in a cyclohexane/buffer batch system. Results shown in percentage appearing in crude reaction mixture analysed by GC.

entry	*reaction time*	*conversion to nitrile **4** / %*
1	15 min	6
2	30 min	13
3	1 h	27
4	2 h	51
5	4 h	79

Table S8: Results of the OxdB-catalysed dehydration of octanal oxime (**3**) in a cyclohexane/buffer batch system. Results shown in percentage appearing in crude reaction mixture analysed by GC. Changed conditions: Addition of Tween 20 (1.3 mg) to aqueous phase.

entry	*reaction time*	*conversion to nitrile **4** / %*
1	5 min	4
2	15 min	15
3	30 min	32
4	1 h	57
5	1 h 45 min	85

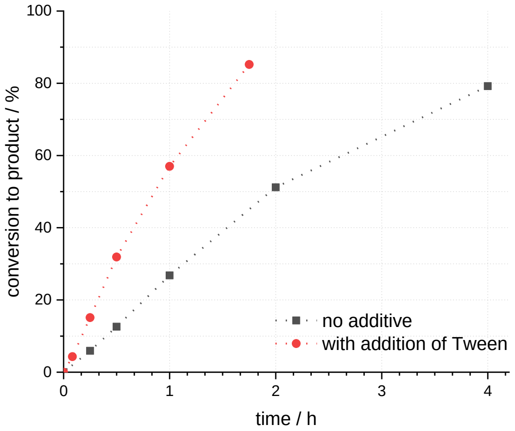

Figure S3: Cyclohexane/buffer batch approach for the OxdB-catalysed dehydration of octanal oxime (**3**) with and without addition of Tween 20.

4.7 Synthesis and isolation of octane nitrile in biphasic systems (batch mode)

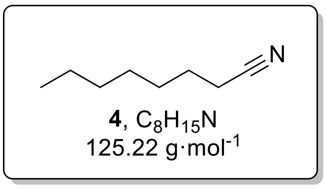

In a 50 mL pear-shaped flask, whole cells containing OxdB* (2 mL of a 333 mg mL^{-1} suspension) were suspended in KP_i buffer (8 µL, 50 mM, pH 7) and Tween 20 (17.4 mg) was added. A solution of octanal oxime (145.1 mg, 1.01 mmol, $c_{organic}$: 101 mM) in cyclohexane (to 10 mL) was added at 30 °C. The reaction mixture was stirred for 3:15 h with a magnetic stirring bar (600 rpm) at 30 °C. The poorly separated suspension was centrifuged (10 000 x g, 1 min) and the phases were separated. The solvent from the organic phase was evaporated to give the desired product (49.4 mg, 39% yield) as colourless liquid. While the aqueous phase was two times extracted with cyclohexane (each 10 mL) and centrifuged (20 000 x g, 20 min). From the combined organic phases, the solvent was removed under reduced pressure and the product **4** obtained as colourless liquid (31.8 mg, 25%). The products were analysed via GC and ^{1}H NMR analysis. The combined product (81.2 mg) was obtained in 64% yield (with >95% purity determined by ^{1}H NMR) and the conversion was determined to be >99%.

GC: Octane nitrile (**4**) 2.4 min, octanal oxime (**3**) 2.7 min.

^{1}H NMR (500 MHz, Chloroform-*d*) δ / ppm = 2.34 (t, *J* = 7.2 Hz, 2H), 1.66 (p, *J* = 7.2 Hz, 2H), 1.55 – 1.40 (m, 2H), 1.39 – 1.25 (m, 8H), 0.90 (t, *J* = 6.8 Hz, 3H).

*This wet-cell mass loading is higher compared to the other reactions since the activity for the freshly prepared whole-cell catalyst was found to be lower. Activities have been compared to a test reaction (see Table S8 Entry) and accordingly adjusted.

4.8 Synthesis of octane nitrile in liquid/liquid segmented flow

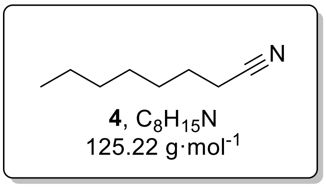

A syringe pump was connected to a Y-mixer (0.5 mm ID), which was connected to a tubular reactor (PFE, 0.8 mm ID, 1 mL). Reaction temperature (30 °C) was controlled by a water bath. For the organic solution, octanal oxime (0.5 mmol, 71.5 mg, $c_{organic}$: 100 mM) was dissolved in cyclohexane (to 5 mL). For the aqueous solution, wet cells containing OxdB (500 µL of a 333 mg·mL^{-1} suspension) were suspended in KP_i buffer (pH 7, 50 mM, to 5 mL). The reaction solution was collected in glass vials containing HCl solution (2 M aq. soln., 0.5 mL) for quenching of the reaction. To start the reaction, the syringe pump was set up (1 mL·h^{-1}, residence time: 30 min). For GC analysis, reaction mixture (20 µL) was diluted with ethyl acetate (100 µL) and analysed via GC.

GC: Octane nitrile (**4**) 2.4 min, octanal oxime (**3**) 2.7 min.

Table S9: Results of the OxdB-catalysed dehydration of octanal oxime (**3**) in cyclohexane/buffer segmented flow. Results shown in percentage appearing in crude reaction mixture analysed by GC. Conditions: Addition of Tween 20 (6.6 mg) to aqueous phase.

entry	*fraction time*	*conversion to nitrile **4** / %*
1	0.5 – 1 h	96
2	1 – 1.5 h	98
3	1.5 – 2.5 h	97
4	2.5 – 3.5 h	95

Table S11: Results of the OxdB-catalysed dehydration of octanal oxime (**3**) in cyclohexane/buffer segmented flow. Results shown in percentage appearing in crude reaction mixture analysed by GC. Conditions: No addition of Tween 20.

entry	*fraction time*	*conversion to nitrile **4** / %*
1	0.5 – 1 h	70
2	1 – 1.5 h	67
3	1.5 – 2 h	67
4	2 – 3.3 h	68
5	3.3 – 3.9 h	68

4.9 Synthesis and isolation of octane nitrile in liquid/liquid segmented flow

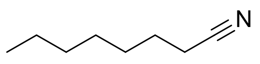

4, $C_8H_{15}N$
125.22 g·mol^{-1}

A syringe pump was connected to a Y-mixer (0.5 mm ID), which was connected to a tubular reactor (PFE, 0.8 mm ID, 1 mL). Reaction temperature (30 °C) was controlled by a water bath. For the organic solution, octanal oxime (1.01 mmol, 144.87 mg, $c_{organic}$: 101 mM) was dissolved in cyclohexane (to 10 mL). For the aqueous solution, wet cells containing OxdB* (2 mL of a 333 mg·mL^{-1} suspension) were suspended in KP_i buffer (pH 7, 50 mM, to 10 mL). Both solutions were transferred into glass syringes and mounted on a syringe pump. To start the reaction, the syringe pump was set up (1 mL·h^{-1}, residence time: 30 min). Two fractions were collected. The first fraction (F1) from 1:09 – 4:39 h run time, and the second (F2) from 4:40 – 8:52 h. After phase separation, the solvent from both volumes was removed to give the product as colourless liquid (F1: 37.2 mg, 89% conversion; F2: 43.1 mg, 94% conversion (determined by GC), 82% crude yield (94% purity determined by ^{1}H NMR)). The product was analysed via GC and ^{1}H NMR.

GC: Octane nitrile (**4**) 2.4 min, octanal oxime (**3**) 2.7 min.

^{1}H NMR (500 MHz, Chloroform-*d*) δ / ppm = 2.34 (t, *J* = 7.2 Hz, 2H), 1.66 (p, *J* = 7.2 Hz, 2H), 1.55 – 1.40 (m, 2H), 1.39 – 1.25 (m, 8H), 0.90 (t, *J* = 6.8 Hz, 3H).

*This wet-cell mass loading is higher compared to the other reactions since the activity for the freshly prepared whole-cell catalyst was found to be lower. Activities have been compared to a test reaction (see Table S8 Entry) and accordingly adjusted.

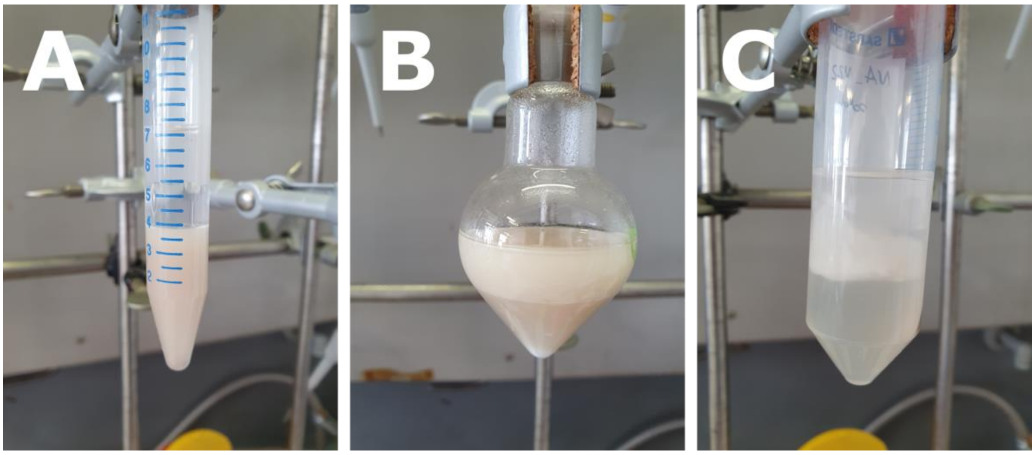

Figure S5: A: Biphasic reaction mixture after flow reaction (without quenching see 4.7); B: Biphasic reaction mixture after batch reaction (see 4.5); biphasic batch reaction mixture after extraction and centrifugation.

4.10 Synthesis of 13-HPOT

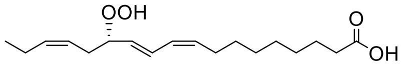

$C_{18}H_{30}O_4$
310.21 g/mol

α-Linolenic acid (**5,** 300 mg, 1.08 mmol) was diluted in ethanol (1 mL). The solution was dissolved in 300 mL ammoniumchloride buffer (100 mM, pH 9) and 14 mL ethanol. Lipoxygenase from *glycine max* (9.12 mg) was dissolved in 1 mL ammoniumchloride buffer (100 mM, pH 9). The reaction was carried out under constant oxygen stream at room temperature. Reaction was controlled *via* TLC. Afterwards the reaction mixture was acidified to pH 2 with hydrochlorid acid (2 M) and extracted twice with MTBE (1:1, *v/v*). Magnesium sulfate was added to remove residual water. The solvent was removed *via* rotary evaporator. 13-HPOT (280 mg, 0.90 mmol, 83%) was isolated as a yellow oil.

^{1}H NMR (500 MHz, CDCl3) δ/ ppm: 6.58 (ddt, *J* = 15.3, 11.1, 1.0 Hz, 1H), 5.99 (t, ^3J = 11.0 Hz, 1H;) 5.58 (dd, ^3J = 8.0 Hz ^4J = 15.2, 1H), 5.53 – 5.47 (m, 2H), 5.33 (dtt, ^3J = 7.3 Hz, ^4J = 10.7, 1.7 Hz, 1H), 4.43 (dt, ^3J = 8.2, 6.6 Hz, 1H), 2.50 – 2.44 (m, 1H), 2.34 (t, ^3J = 7.4 Hz, 2H), 2.32 – 2.28 (m, 1H), 2.18 (dtd, ^3J =7.4 Hz, ^4J =1.5, 15.0 Hz, 2H), 2.05 (pd, ^3J = 7.4 Hz, ^4J = 1.5 Hz, 2H), 1.63 (q, ^3J = 7.3 Hz, 3H), 1.25 (td, ^3J = 7.1, 4.6 Hz), 0.96 (t, ^3J = 7.5 Hz, 3H).

^{13}C NMR (500 MHz, CDCl3) δ/ ppm: 178.46, 134.53, 134.26, 130.42, 130.36, 127.68, 123.22, 86.36, 33.89, 30.76, 29.42, 29.00, 28.94, 27.80, 24.77, 20.85, 14.25.

EI-MS [m/z]: 333.26 $[M+Na]^+$349.17 $[M+K]^+$

The successful synthesis could be proven by literature.

4.11 Synthesis of 12-OPDA in batch (analytical scale)

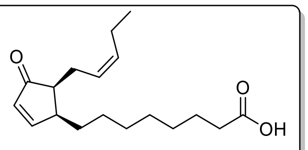

$C_{18}H_{28}O_3$
292.42 g/mol

Buffer (100 mM, pH 8) was saturated with oxygen. *E.coli* BL21CodonPlus(DE3)-RIL containing AtAOS and AtAOC2 (10/20/30 mg) was diluted in 1 mL saturated in buffer. 13-HPOT (7.50 mg, 0.02 mmol) was dissolved in 1 mL solvent (with or without the addition of 1 vol% Tween® 20). The reaction was done at room temperature, at 400 rpm and for 30 minutes. Afterwards the reaction was quenched with 2 M HCl (500 µL). The supernatant has been removed and the aqueous was extracted with dichloromethane (1 mL). The solvent was removed *in vacuo* and analyzed by the means of ^{1}H NMR-spectroscopy.

Table S12: Reaction conditions and results for 12-OPDA (**8**) synthesis in batch.

Buffer / Cosolvent	*Whole cells / mg*	*Stirrer / rpm*	*Conv. / %*
**NaPi, pH 8**	30	400	99
**NH_4Cl, pH 8**	30	400	35
**NH_4Cl, pH 9**	30	400	99
**5x Cellmass**	30	400	99
**Cyclohexane**	30	400	50
**Cyclohexane + 1 vol% Tween® 20**	30	400	74
**Cyclohexane + 10% MTBE**	30	400	85
**Cyclohexane + 50% MTBE**	30	400	31
**Isooctane**	30	400	99
**Ethylacetate**	30	400	14
**Cyclohexane + 10% MTBE**	10	400	8
**Cyclohexane + 10% MTBE + 1% Tween**	10	400	8
**Isooctane**	10	400	9
**Isooctane + 1% Tween**	10	400	9
**Isooctane + 1% Tween**	20	400	34
**Isooctane + 1% Tween**	20	1400	0

^{1}H NMR (500 MHz, $CDCl_3$) δ/ppm = 7.73 (dd, ^3J = 6.0, ^4J = 2.7 Hz, 1H), 7.60 (d, ^3J = 5.7 Hz, 1H), 6.18 (dd, ^3J = 5.9, ^4J = 1.7 Hz, 1H), 6.12 (d, ^3J = 5.9 Hz, 1H), 5.46 – 5.32 (m, 2H), 2.97 (ddt, ^3J = 5.9, 10.8, 7.6, ^4J = 3.6 Hz, 1H), 2.50 (dt, ^3J = 15.3, 5.4 Hz, 1H), 2.47 – 2.41 (m, 1H), 2.35 (t, ^3J = 7.5 Hz, 2H), 2.17 – 2.10 (m, 1H), 2.06 (d, ^3J = 7.5 Hz, 2H), 1.72 (td, ^3J = 11.1, 5.0 Hz, 1H), 1.63 (q, ^3J = 7.2 Hz, 3H), 1.32 (q, ^3J = 7.1, 5.9 Hz, 8H), 1.15 (dtd, J = 14.3, 9.6, ^4J = 4.5 Hz, 1H), 0.97 (t, ^3J = 7.5 Hz, 3H).

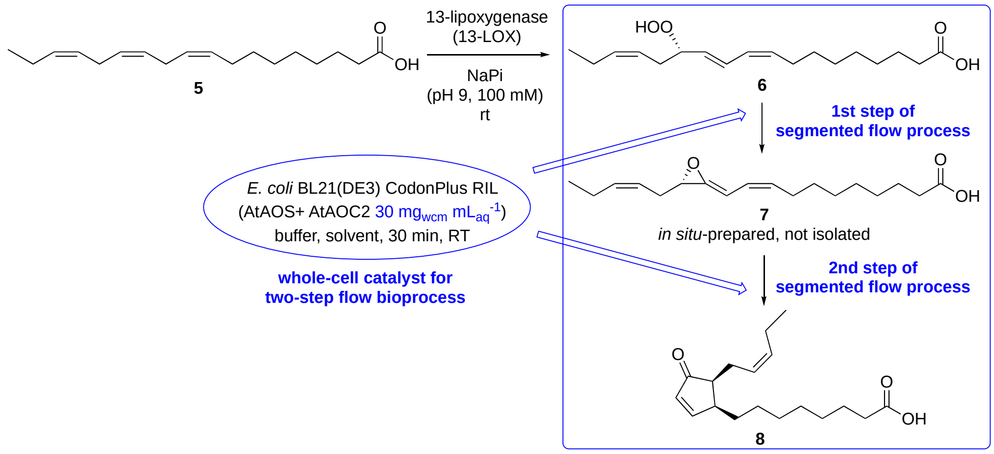

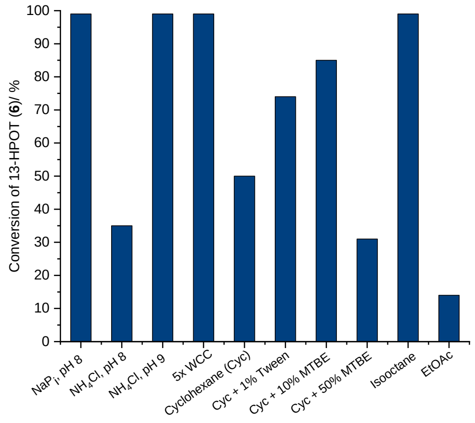

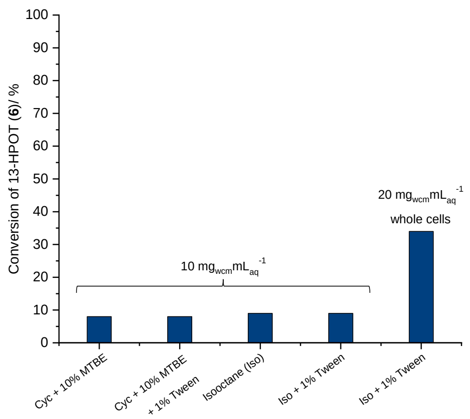

Figure S6. Synthesis of 12-OPDA (**8**) starting from 13-HPOT (**6**) (left) with a catalyst loading of 30 mg_{wcm} mL_{aq}^{-1} using a variety of buffers and solvents, (right) selected solvents with 20 and 10 mg_{wcm} mL_{aq}^{-1} whole cells.

The successful synthesis has been confirmed by comparison of the analytical data with those from literature.

4.12 Synthesis of 12-OPDA in batch (preparative scale)

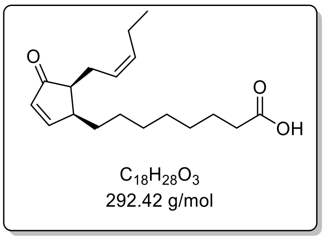

NaPi-buffer (100 mM, pH 8) was saturated with oxygen. *E.coli* BL21CodonPlus(DE3)-RIL containing AtAOS and AtAOC2 (200 mg) was diluted in 1 mL saturated in buffer. 13-HPOT (53.0 mg, 0.17 mmol) was dissolved in 5 mL isooctane with addition of 1 vol% Tween® 20. The reaction was done at room temperature, at 400 rpm and for 30 minutes. Afterwards the reaction was quenched with 2 M HCl (500 µL) and centrifuged (10.000x g, 5 min). The supernatant has been removed and the aqueous was extracted with dichloromethane (5 mL). The solvent was removed *in vacuo* and analyzed by the means of ^{1}H NMR-spectroscopy. The crude product **8** was isolated with a yield of 36% (19 mg, 0.07 mmol).

^{1}H NMR (500 MHz, $CDCl_3$) δ/ppm = 7.73 (dd, ^3J = 6.0, ^4J = 2.7 Hz, 1H), 7.60 (d, ^3J = 5.7 Hz, 1H), 6.18 (dd, ^3J = 5.9, ^4J = 1.7 Hz, 1H), 6.12 (d, ^3J = 5.9 Hz, 1H), 5.46 – 5.32 (m, 2H), 2.97 (ddt, ^3J = 5.9, 10.8, 7.6, ^4J = 3.6 Hz, 1H), 2.50 (dt, ^3J = 15.3, 5.4 Hz, 1H), 2.47 – 2.41 (m, 1H), 2.35 (t, ^3J = 7.5 Hz, 2H), 2.17 – 2.10 (m, 1H), 2.06 (d, ^3J = 7.5 Hz, 2H), 1.72 (td, ^3J = 11.1, 5.0 Hz, 1H), 1.63 (q, ^3J = 7.2 Hz, 3H), 1.32 (q, ^3J = 7.1, 5.9 Hz, 8H), 1.15 (dtd, J = 14.3, 9.6, ^4J = 4.5 Hz, 1H), 0.97 (t, ^3J = 7.5 Hz, 3H).

4.13 Synthesis of 12-OPDA in segmented flow (analytical scale)

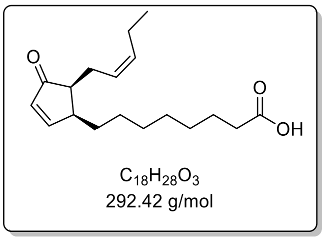

NaPi-buffer (100 mM, pH 8) was saturated with oxygen. *E.coli* BL21CodonPlus(DE3)-RIL containing AtAOS and AtAOC2 (20 mg_{wcm} ml_{aq}^{-1}) was diluted in 5 mL saturated NaPi-buffer (100 mM, pH 8) and 1 vol% Tween® 20. The reaction solution was transferred into a syringe (5 mL, S.G.E., gas tight, 10.3 mm ID). A second solution of 13-HPOT (7.50 mg, 0.02 mmol) was dissolved in 5 mL isooctane and transferred into a syringe (5 mL, S.G.E., gas tight, 10.3 mm ID). The feed of the two solutions (each Q: 1mL h^{-1} pumped with a syringe pump) were mixed in a Y mixer (0.5 mm ID). The segments were led into a tubular reactor (PFE, 0.8 mm ID, 1 mL). To start the reaction, the syringe pump was set up (1 mL·h^{-1}, τ = 30 min). Fractions were collected and in a glass vial containing 2 M HCl (1 mL). The supernatant has been removed and the aqueous phase was extracted with dichloromethane (1 mL). The solvent was removed in vacuo and analyzed by the means of ^{1}H NMR.

Table S13: Results of the AtAOS/AOC2 catalysed reaction towards 12-OPDA (**8**) in isooctane/buffer segmented flow. Results shown in percentage appearing in crude reaction mixture analysed by ^{1}H NMR-spectroscopy.

Entry	*fraction time/h*	*Conversion to **8**/ %*
1	0.5 – 1.5	>99
2	1.5 – 2.5	>99
3	2.5 – 3.5	>99
4	3.5 – 4.5	>99
5	4.5 – 5.5	>99

^{1}H NMR (500 MHz, $CDCl_3$) δ/ppm = 7.73 (dd, ^3J = 6.0, ^4J = 2.7 Hz, 1H), 7.60 (d, ^3J = 5.7 Hz, 1H), 6.18 (dd, ^3J = 5.9, ^4J = 1.7 Hz, 1H), 6.12 (d, ^3J = 5.9 Hz, 1H), 5.46 – 5.32 (m, 2H), 2.97 (ddt, ^3J = 5.9, 10.8, 7.6, ^4J = 3.6 Hz, 1H), 2.50 (dt, ^3J = 15.3, 5.4 Hz, 1H), 2.47 – 2.41 (m, 1H), 2.35 (t, ^3J = 7.5 Hz, 2H), 2.17 – 2.10 (m, 1H), 2.06 (d, ^3J = 7.5 Hz, 2H), 1.72 (td, ^3J = 11.1, 5.0 Hz, 1H), 1.63 (q, ^3J = 7.2 Hz, 3H), 1.32 (q, ^3J = 7.1, 5.9 Hz, 8H), 1.15 (dtd, J = 14.3, 9.6, ^4J = 4.5 Hz, 1H), 0.97 (t, ^3J = 7.5 Hz, 3H).

The successful synthesis has been confirmed by comparison of the analytical data with those from literature.

4.14 Synthesis of 12-OPDA in segmented flow (preparative scale)

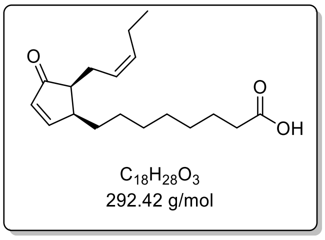

NaPi-buffer (100 mM, pH 8) was saturated with oxygen. *E.coli* BL21CodonPlus(DE3)-RIL containing AtAOS and AtAOC2 (200 mg_{wcm} ml_{aq}^{-1}) was diluted in 5 mL saturated NaPi-buffer (100 mM, pH 8) and 1 vol% Tween® 20. The reaction solution was transferred into a syringe (5 mL, S.G.E., gas tight, 10.3 mm ID). A second solution of 13-HPOT (53.0 mg, 0.17 mmol) was dissolved in 5 mL isooctane and transferred into a syringe (5 mL, S.G.E., gas tight, 10.3 mm ID). The feed of the two solutions (each Q: 1mL h^{-1} pumped with a syringe pump) were mixed in a Y mixer (0.5 mm ID). The segments were led into a tubular reactor (PFE, 0.8 mm ID, 1 mL). To start the reaction, the syringe pump was set up (0.5 mL·h^{-1}, τ = 60 min). Fractions were collected and in a glass vial containing 2 M HCl (1 mL). The supernatant has been removed and the aqueous was extracted with dichloromethane (1 mL). The organic phases of all fractions were combined and the solvent was removed *in vacuo* and analyzed by the means of ^{1}H NMR-spectroscopy. The crude product **5** was isolated with a yield of 65% (44 mg, 0.15 mmol).

^{1}H NMR (500 MHz, $CDCl_3$) δ/ppm = 7.73 (dd, ^3J = 6.0, ^4J = 2.7 Hz, 1H), 7.60 (d, ^3J = 5.7 Hz, 1H), 6.18 (dd, ^3J = 5.9, ^4J = 1.7 Hz, 1H), 6.12 (d, ^3J = 5.9 Hz, 1H), 5.46 – 5.32 (m, 2H), 2.97 (ddt, ^3J = 5.9, 10.8, 7.6, ^4J = 3.6 Hz, 1H), 2.50 (dt, ^3J = 15.3, 5.4 Hz, 1H), 2.47 – 2.41 (m, 1H), 2.35 (t, ^3J = 7.5 Hz, 2H), 2.17 – 2.10 (m, 1H), 2.06 (d, ^3J = 7.5 Hz, 2H), 1.72 (td, ^3J = 11.1, 5.0 Hz, 1H), 1.63 (q, ^3J = 7.2 Hz, 3H), 1.32 (q, ^3J = 7.1, 5.9 Hz, 8H), 1.15 (dtd, J = 14.3, 9.6, ^4J = 4.5 Hz, 1H), 0.97 (t, ^3J = 7.5 Hz, 3H).

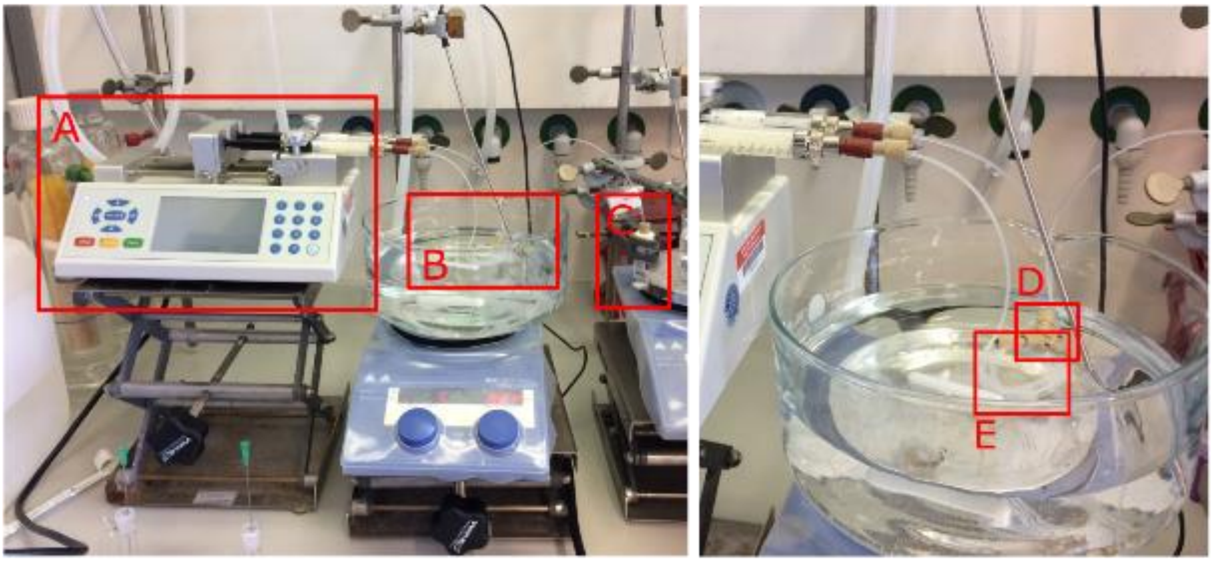

Figure S7: Photo of the reaction setup. A: syringe pump with aqueous whole-cell catalyst and organic substrate solution, B: reactor and mixer, C: vial containing quenching solution for collecting the product, D: Y-mixer (PEEK), E: PTFE coil reactor (0.8 mm ID, 1 mL).

5 ^{1}H NMR Chromatograms

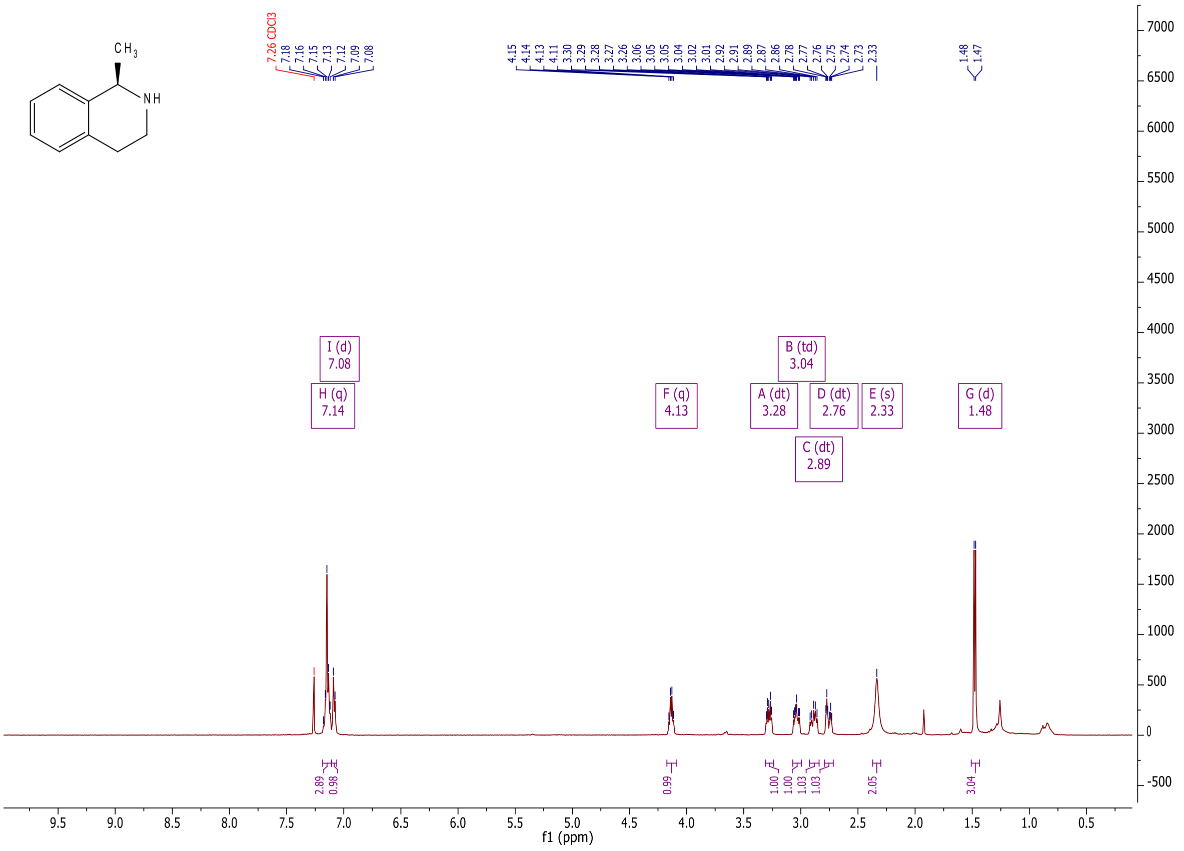

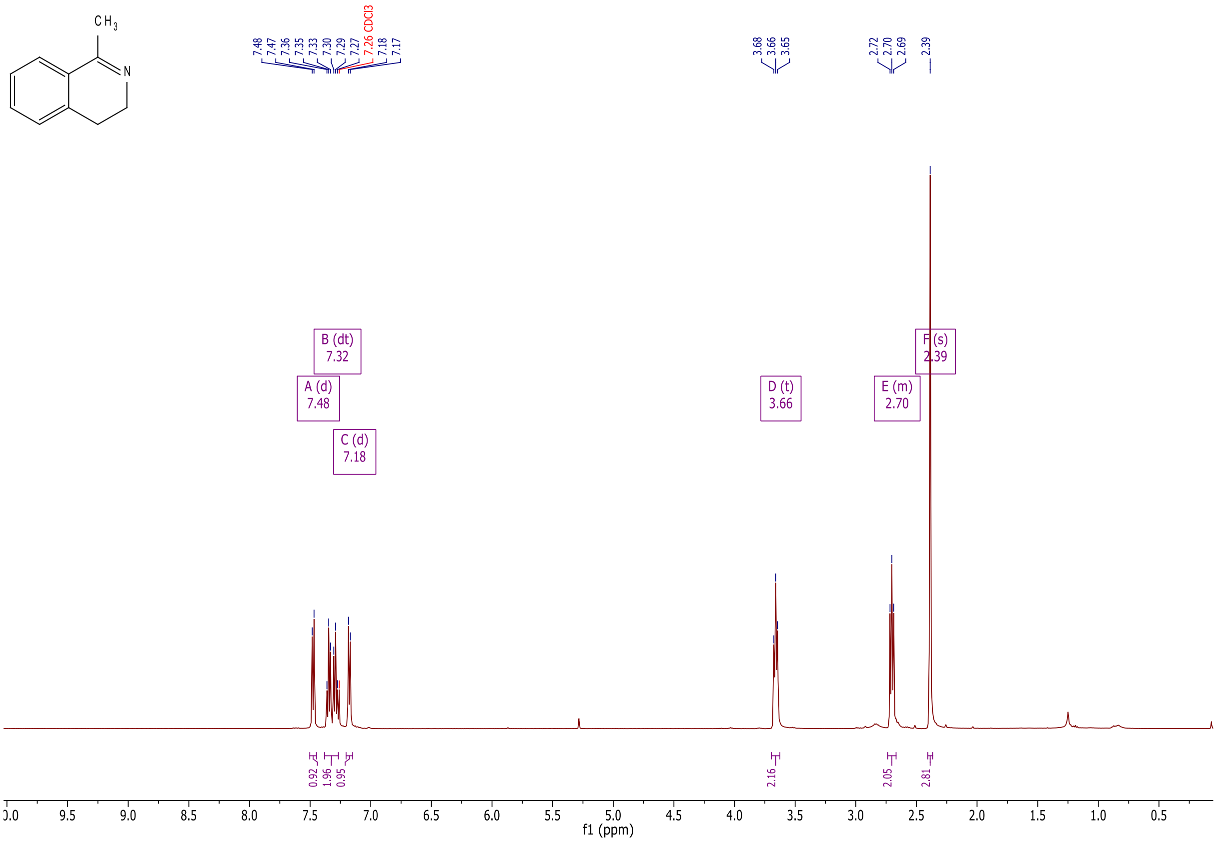

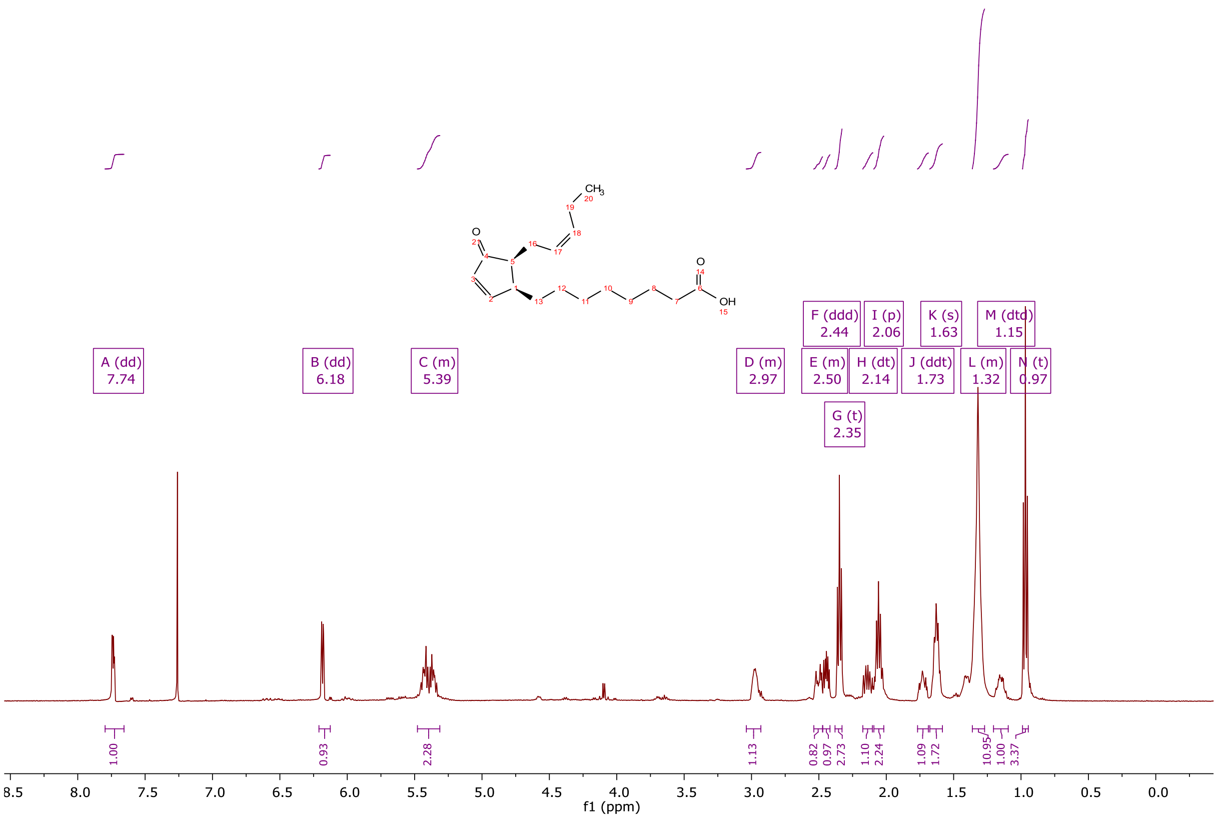

A (dd) 7.74
B (dd) 6.18
C (m) 5.39
D (m) 2.97
E (m) 2.50
F (ddd) 2.44
G (t) 2.35
H (dt) 2.14
I (p) 2.06
J (ddt) 1.73
K (s) 1.63
L (m) 1.32
M (dtd) 1.15
N (t) 0.97
f1 (ppm)

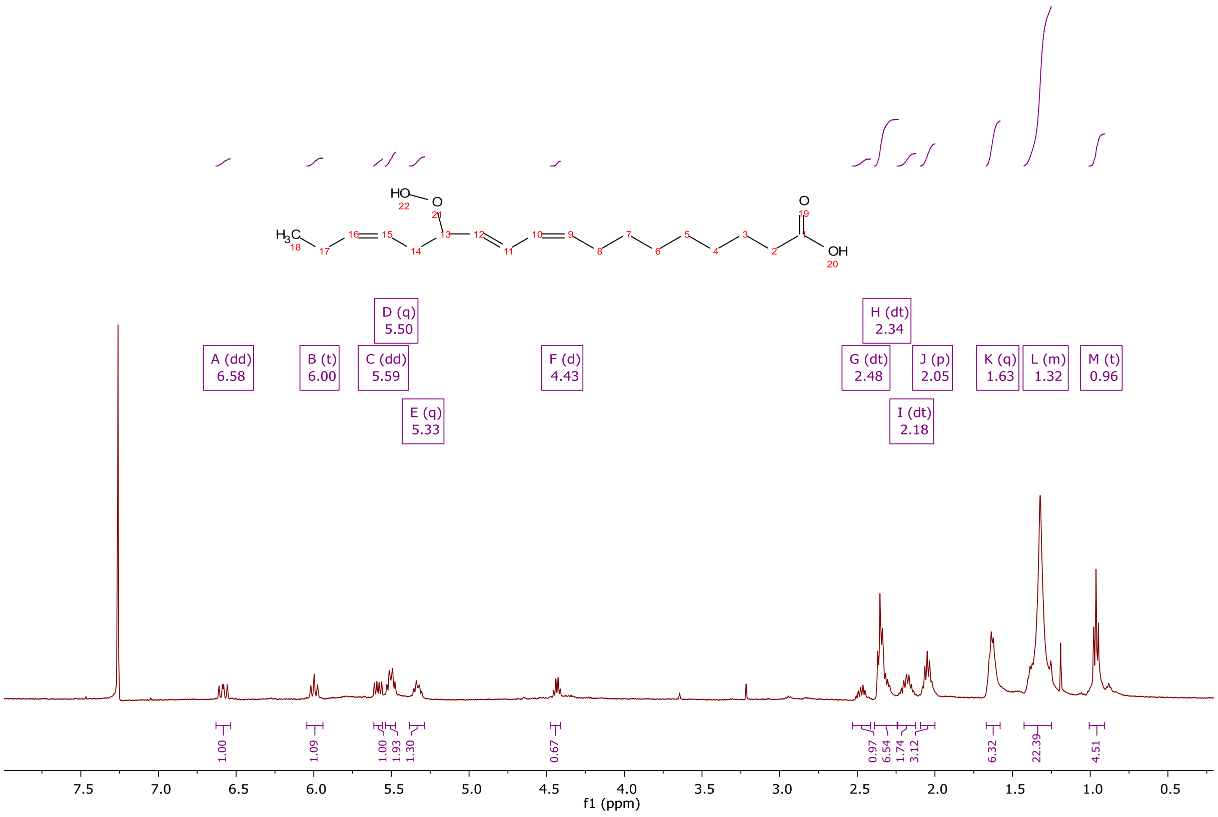

A (dd) 6.58
B (t) 6.00
C (dd) 5.59
D (q) 5.50
E (q) 5.33
F (d) 4.43
G (dt) 2.48
H (dt) 2.34
I (dt) 2.18
J (p) 2.05
K (q) 1.63
L (m) 1.32
M (t) 0.96
f1 (ppm)

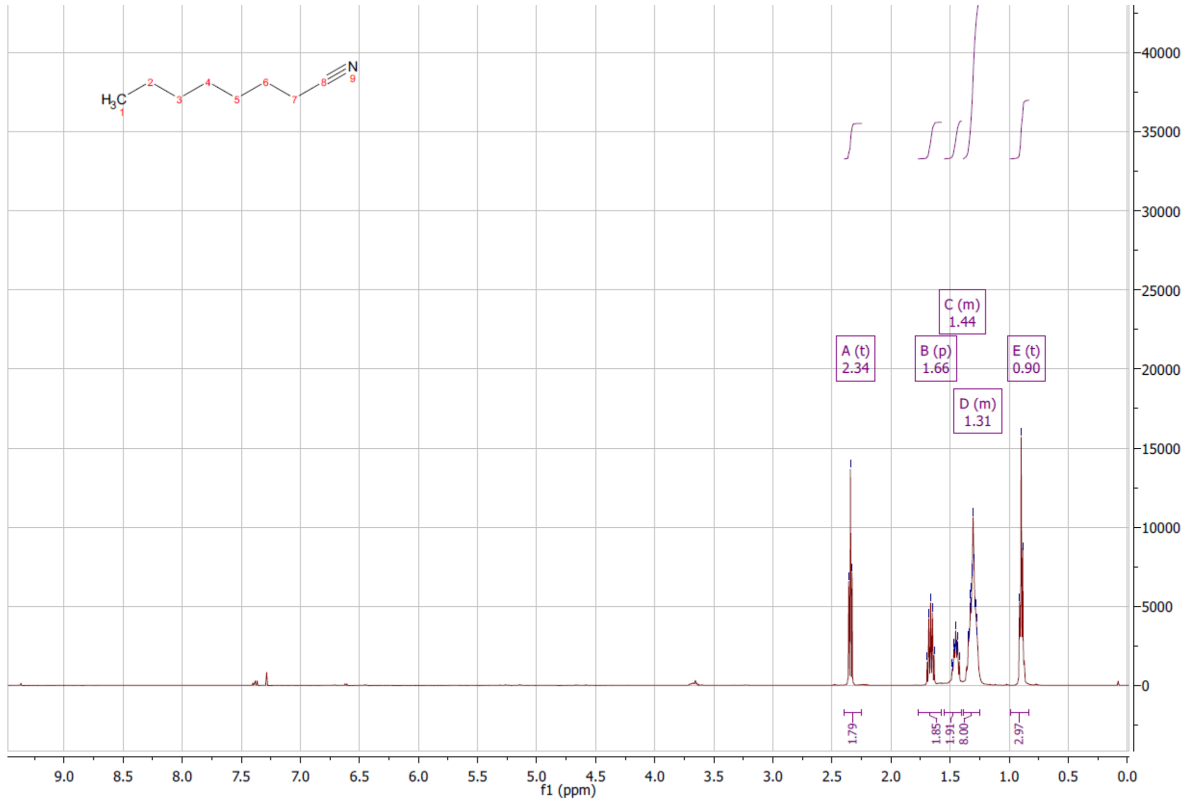

6 References

1. N. Zumbrägel, D. Wetzl, H. Iding, H. Gröger, *Heterocycles* **2017**, *95*, 1261-1271.

2. M. Biermann, H. Gruß, W. Hummel, H. Gröger, *ChemCatChem* **2016**, *8*, 895-899.

3. D. Wetzl, M. Berrera, N. Sandon, D. Fishlock, M. Ebeling, M. Müller, S. Hanlon, B. Wirz, H. Iding, *ChemBioChem* **2015**, *16*, 1749-1756.

4. A. Hinzmann, N. Adebar, T. Betke, M. Leppin, H. Gröger, *Eur. J. Org. Chem.* **2019**, 6911-6916.

5. I. Stenzel, B. Hause, O. Miersch, T. Kurz, H. Maucher, H. Weichert, J. Ziegler, I. Feussner, C. Wasternack, *Plant Mol. Biol.* **2003**, *51*, 895-911.

6. j. Scholz, F. Brodhun, E. Hornung, C. Herrfurth, M. Stumpe, A. K. Beike, B. Faltin, W. Frank, R. Reski, I. Feussner, *BMC Plant Biol.* **2012**, *12*, 228-243.
